# A General Strategy for Food Traceability and Authentication Based on Assembly‐Tunable Fluorescence Sensor Arrays

**DOI:** 10.1002/advs.202309259

**Published:** 2024-05-17

**Authors:** He Cheng, Tianyue Liu, Jingsheng Tian, Ruixuan An, Yao Shen, Mingxi Liu, Zhiyi Yao

**Affiliations:** ^1^ Beijing Laboratory of Food Quality and Safety College of Food Science and Nutritional Engineering China Agricultural University Beijing 100083 China

**Keywords:** fluorescent probe, food traceability and authentication, sensor array, tunable assembly

## Abstract

Food traceability and authentication systems play an important role in ensuring food quality and safety. Current techniques mainly rely on direct measurement by instrumental analysis, which is usually designed for one or a group of specific foods, not available for various food categories. To develop a general strategy for food identification and discrimination, a novel method based on fluorescence sensor arrays is proposed, composed of supramolecular assemblies regulated by non‐covalent interactions as an information conversion system. The stimuli‐responsiveness and tunability of supramolecular assemblies provided an excellent platform for interacting with various molecules in different foods. In this work, five sensor arrays constructed by supramolecular assemblies composed of pyrene derivatives and perylene derivatives are designed and prepared. Assembly behavior and sensing mechanisms are investigated systematically by spectroscopy techniques. The traceability and authentication effects on several kinds of food from different origins or grades are evaluated and verified by linear discriminant analysis (LDA). It is confirmed that the cross‐reactive signals from different sensor units encompassing all molecular interactions can generate a unique fingerprint pattern for each food and can be used for traceability and authentication toward universal food categories with 100% accuracy.

## Graphical abstract

A general strategy for food traceability and authentication based on supramolecular fluorescence sensor arrays regulated by non‐covalent interactions is designed. The sensor arrays composed of supramolecular assemblies are an information conversion system that transforms the overall composition of food into a general signal and generate a unique fingerprint pattern for each food.

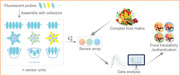

## 1 Introduction

Food traceability and authentication systems, aiming to track agri‐food products from “field to table”, play an important role in ensuring food quality and safety.^[^ ^1^ ^]^ Traceability in the food supply chain makes it possible to identify the source of contaminated food so that problematic food can be quickly recalled or withdrawn from the market,^[^ ^2^ ^]^ thereby minimizing the risk of foodborne disease transmission and safeguarding food quality and public health. On the other hand, as economic globalization advances and human civilization progresses, many foods or agricultural products have become renowned for their specific geographic origins. These products with geographic indications offer a more delightful taste and greater nutritional benefits.^[^ ^3^ ^]^ Subsequently, profit‐driven food fraud incidents have occurred frequently, which seriously damaged the interests of consumers. In this case, traceability and authentication technology can increase the transparency of the food chain, thus effectively reducing the risk of food adulteration and counterfeiting.^[^ ^4^ ^]^

Current techniques for food traceability and authentication mainly rely on genomics/proteomics,^[^ ^5^ ^]^ chromatography/mass spectrometry,^[^ ^6^ ^]^ elemental profiling,^[^ ^7^ ^]^ nuclear magnetic resonance (NMR),^[^ ^8^ ^]^ sensory analysis^[^ ^9^ ^]^ and spectroscopic methods (such as UV–vis spectroscopy,^[^ ^10^ ^]^ infrared spectroscopy,^[^ ^11^ ^]^ fluorescence spectroscopy,^[^ ^12^ ^]^ and Raman spectroscopy^[^ ^13^ ^]^). Although relatively accurate results can be obtained from these methods, further application of some methods is limited because of laboratory‐based sophisticated instruments and complex, time‐consuming operations.^[^ ^14^ ^]^ More importantly, existing traceability/authentication methods are usually designed for one or a group of specific foods, not available for different food categories. Hence, it is of great importance to develop a general and efficient strategy for food traceability and authentication in the face of various foods with vast growing areas, diverse growing environments, and complex supply chain systems.

In fact, the essential differences between different categories of foods are their own composition such as proteins, lipids, carbohydrates, vitamins, minerals, and other specific components related to this kind of food. Direct measurement of each component is certainly a cumbersome and inefficient way for traceability and authentication. To convert the overall composition of food into a general signal and generate a unique fingerprint pattern for each food, we first attempt to construct a sensor array using fluorescent supramolecular assemblies regulated by non‐covalent interactions as an information conversion system for food identification and discrimination. Sensor array with a set of sensor units is a multi‐sensing technique that is achieved by collecting responses from differential interactions between analytes and sensors.^[^ ^15^ ^]^ It has been widely applied to distinguish various molecules, such as ions,^[^ ^16^ ^]^ amino acids,^[^ ^17^ ^]^ proteins,^[^ ^18^ ^]^ pathogens,^[^ ^19^ ^]^ and so forth. Supramolecular assemblies bridge the gap between the molecular scale and the macroscopic one in terms of structure and performance. They can be constructed by various building blocks and assembly modes, thereby providing diverse sensing elements with tunable type and quantity. Their dynamic and reversible nature endows them with stimuli‐responsive and tunable interactions under microenvironmental conditions.^[^ ^20^ ^]^ Given the fact that non‐covalent interactions are ubiquitous^[^ ^21^ ^]^ and these interactions in the binding assay of analyte are tunable such as electrostatic, hydrophobic, hydrogen bond, π─π, host–guest, and other non‐covalent interactions, it is expected that various molecules in different foods can interact with assemblies to cause different fluorescent signals. These cross‐reactive signals from different sensor units encompassing all molecular interactions can generate a unique fingerprint pattern for each food to realize identification and discrimination.^[^ ^22^ ^]^ Consequently, the assembly‐tunable fluorescence sensor arrays have the potential to achieve traceability and authentication of various foods.

**Scheme 1:**
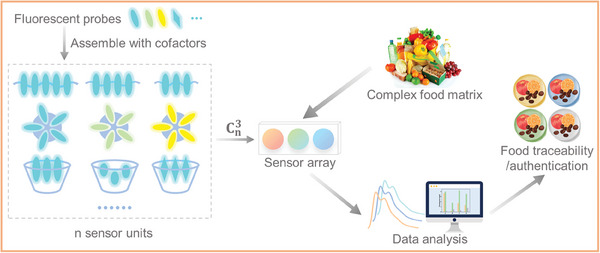
Schematic illustration of supramolecular fluorescence sensor arrays for food traceability and authentication.

In this work, we take various sensor arrays composed of assemblies based on pyrene and perylene derivatives as examples to conduct traceability and authentication on several different kinds of foods to illustrate the feasibility and universality of this strategy. As fluorescent probes, functionalized pyrenes (Pys) and perylenes (Pls) possess stable chemical properties and excellent optical sensing performance and have been widely used for sensing biomacromolecules, small organic molecules, ions and so on.^[^ ^23^ ^]^ In our strategy, a variety of supramolecular assemblies based on fluorescent probes by combining with different types of cofactors such as polyelectrolytes, amphiphiles, macrocyclic molecules, and so on through non‐covalent interactions were depicted in **Scheme** **1**. Each assembly as a sensing unit can generate one signal toward each food and any three sensor units can build a sensor array to produce a signal combination, thereby achieving pattern recognition of the food. Thus, this scheme provides diverse sensor arrays that can cater to the complex and varied requirements of food analysis. To the best of our knowledge, this is the first general strategy for food traceability and authentication based on assembly‐tunable fluorescence sensor arrays. Detailed mechanisms and applications of this strategy will be discussed in the following sections.

## 2 Results and Discussion

### 2.1 Construction of Sensor Arrays and Exploration of Sensing Mechanisms

**Figure 1:**
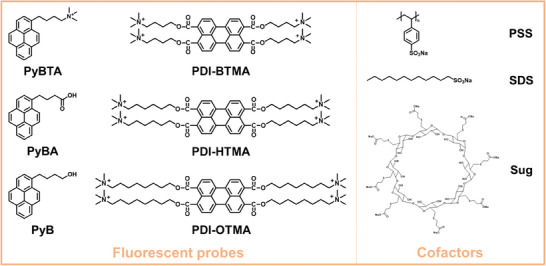
Chemical structures of PyBTA, PyBA, PyB, PDI‐BTMA, PDI‐HTMA, PDI‐OTMA, PSS, SDS, and Sug.

In general, fluorescent supramolecular assemblies consist of fluorescent probes and cofactors through non‐covalent interactions. In view of that the main components of food are lipids, carbohydrates, proteins, vitamins, minerals, and other specific substances, some preliminary considerations concerning the choice of the sensing unit are discussed as follows. Lipids usually have typical long carbon chains and tend to assemble with hydrophobic compounds; carbohydrates and proteins as biological macromolecules possess multiple action sites and they are prone to promote the formation of polyelectrostatic interactions and multi‐hydrogen bonds; structural characteristics of vitamins are aromatic and heteroaromatic rings which enable the interactions of π─π stacking; minerals containing various inorganic ions prefer to form supramolecular complex driven by electrostatic and coordination interactions. According to the above, six fluorescent probes of pyrenyl and perylenyl derivatives and three cofactors with the potential to non‐covalently interact with various food molecules were selected, including *N, N, N*‐trimethyl‐4‐(pyrene‐1‐butyl)‐ammonium bromide (PyBTA), 1‐pyrenebutyric acid (PyBA), 1‐pyrenebutanol (PyB), 3,4,9,10‐tetra‐(4‐trimethylammoniobutyloxy‐carbonyl)‐perylene (PDI‐BTMA), 3,4,9,10‐tetra‐(4‐trimethylaminohexyloxy‐carbonyl)‐perylene (PDI‐HTMA), 3,4,9,10‐tetra‐(4‐trimethylaminooctyloxy‐carbonyl)‐perylene (PDI‐OTMA), poly(sodium 4‐styrenesulfonate) (PSS), sodium dodecyl sulfate (SDS), and sugammadex sodium (Sug) (**Figure** **1**). They were utilized to construct a small sensing unit library, serving as examples to elucidate our strategy.

**Figure 2:**
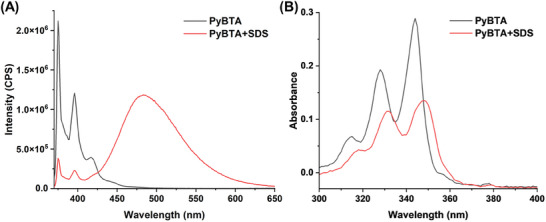
Fluorescence and absorption spectra of PyBTA before and after assembly with SDS in HEPES buffer (10 mm, pH = 7), *λ*_ex_ (excitation wavelength) = 350 nm.

First, the assembly mechanism of PyBTA‐SDS complex was systematically investigated. i) The formation of the PyBTA‐SDS supramolecular assembly was initially demonstrated by absorption and emission spectra (**Figure** **2**A). It exhibited two emission peaks around 375 and 396 nm, representing its light‐emitting monomer form.^[^ ^24^ ^]^ When SDS was added, an emission band centered at 485 nm was observed, indicating the aggregation and generation of pyrene excimer.^[^ ^25^ ^]^ In the absorption spectra (Figure 2B), the positions of 0–0 transitions (≈345 nm) in PyBTA‐SDS supramolecular assembly were slightly bathochromic with respect to those of PyBTA alone, indicating that SDS induced PyBTA aggregation.^[^ ^26^ ^]^ ii) TEM images of PyBTA in the absence and presence of SDS were also collected in Figure S4 (Supporting Information). Larger aggregates with a size of about 100 nm could be observed when PyBTA and SDS coexisted, further confirming the assembly of PyBTA and SDS. iii) To further explain the molecular mechanism of PyBTA excimer induced by SDS, the fluorescence spectra of PyBTA in different solvents were first compared in Figure S5A (Supporting Information). It can be seen that with the decrease of solubility (a good solvent, chloroform>a poor solvent, ether), the emission at 397 nm (assigned to the monomer of PyBTA) gradually decreased and the emission at 485 nm (assigned to the excimer of PyBTA) gradually increased, implying aggregation induced the formation of the excimer. In addition, the fluorescence spectra of PyBTA in solution and in solid state were compared in Figure S5B (Supporting Information). It showed a strong monomer emission band of PyBTA in solution, whereas a broadband excimer emission was observed in the solid‐state, very similar to that of PyBTA‐SDS. This result further supported that the broadband green emission of PyBTA should be attributed to the excimer induced by SDS. iv) The driving force of PyBTA‐SDS assemblies could be explored by comparing the structural analogs of PyBTA and SDS respectively. In Figure S6A (Supporting Information), negatively charged SDS could not cause the fluorescence change of negatively charged 1‐pyrenebutyric acid (PyBA) that is a structural analog of PyBTA. On the other hand, sodium 1‐octanesulfonate (SOS) which is less hydrophobic than SDS, could not induce the excimer emission of PyBTA (Figure S6B, Supporting Information). These results suggested that electrostatic and hydrophobic interactions were the dominant factors in the PyBTA‐SDS supramolecular assembly.

To address the sensing mechanism of PyBTA‐SDS supramolecular assembly toward analytes, various influencing factors on the disassembly of PyBTA‐SDS were systematically investigated in Figure S7 (Supporting Information). It can be seen that solvents (microenvironment polarity), temperatures (weakening non‐covalent interactions), cations and anions with different charges (electrostatic interaction), molecules with different carbon chain lengths (hydrophobic interaction) and different aromatic ring structures (π‐stacking interaction) can all cause distinct changes in the fluorescence signal of PyBTA‐SDS, implying these factors can all induce the disassembly of PyBTA‐SDS at a certain extent. Based on the above experimental results and chemical structures of the analytes, one can conclude the molecular mechanism behind the disassembly of PyBTA‐SDS could be attributed to the effects on non‐covalent interactions (electrostatic, hydrophobic, and π‐stacking, etc.) of PyBTA‐SDS induced by external stimulus. It also confirmed the versatility and sensitivity of the fluorescent supramolecular assembly with its stimuli‐responsive and tunable nature. Since food components are varied and non‐covalent interactions are ubiquitous, the addition of food samples will certainly influence the fluorescence signal of PyBTA‐SDS through various non‐covalent interactions. Hence, a fluorescent supramolecular assembly could convert the overall composition of food into a general signal and generate a unique fingerprint pattern for each food.

**Figure 3:**
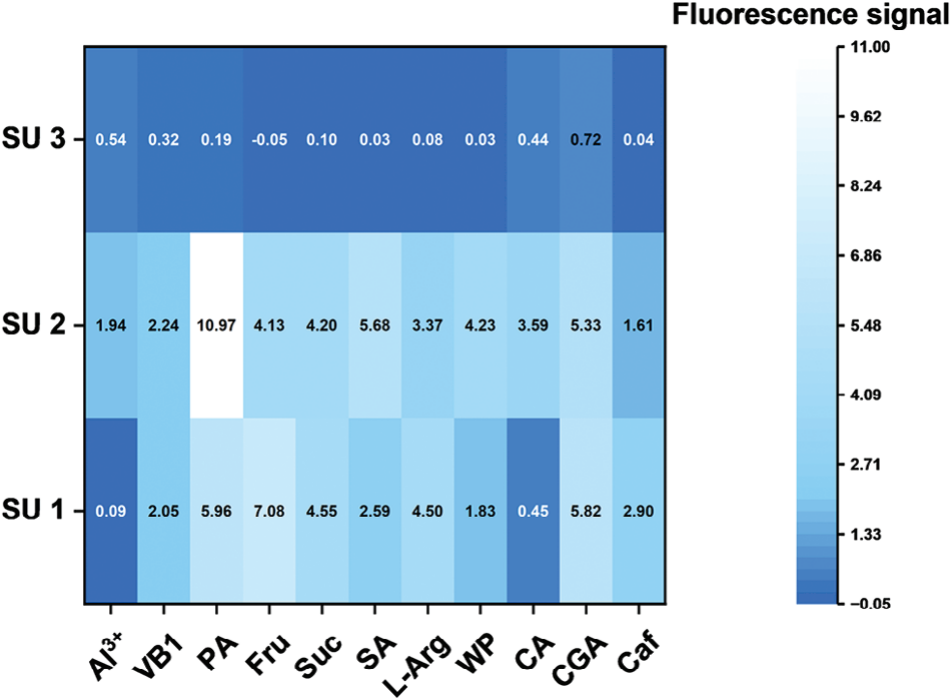
Fluorescence signal heatmap of sensor array 1 interacting with different molecules in HEPES buffer (10 mm, pH = 7), c(Al^3+^) = c(VB1) = c(L‐Arg) = c(Caf) = 0.5 mm, c(PA) = 0.05 mm, c(Fru) = c(Suc) = 50 mm, c(SA) = 10 µg mL^−1^, c(WP) = 1.5 µg mL^−1^, c(CA) = 5 mm, c(CGA) = 0.1 mm. The fluorescence signal is explained in the Experimental Section of Supporting Information.

Based on the above discussion, sensor array 1 was constructed by PyBTA assembled with PSS, SDS, and Sug to further demonstrate the detection mechanism of supramolecular fluorescence sensor arrays. We collected the fluorescence responses of sensor array 1 toward 11 different types of common food molecules and presented a heatmap in **Figure** **3**, including Al^3+^, vitamin B1 (VB1), palmitic acid (PA), fructose (Fru), sucrose (Suc), sodium alginate (SA), l‐arginine (l‐Arg), whey protein (WP), citric acid (CA), chlorogenic acid (CGA), and caffeine (Caf). It can be seen that one sensor unit has different fluorescence responses to different analytes and one analyte causes different fluorescence responses of different sensor units, thereby generating a unique fluorescence signal pattern. Besides categories of food components, we also examined the fluorescence response of sensor array 1 to the same food composition at different concentrations (Figure S8, Supporting Information). It is obvious that distinct fluorescence responses were obtained. These results indicated that for any given food, different categories and concentrations of their own components would make supramolecular fluorescence sensor arrays output different fluorescence signal combinations, thereby leading to differentiation and identification.

Since fluorescent supramolecular assemblies in our strategy can vary with different elements, molecular ratios, and microenvironments of supramolecular assemblies, diverse supramolecular fluorescence sensor arrays can be constructed even with a limited set of molecules. This diversity provides more selectivity and greater potential for traceability and authentication of various foods. To assess the effectiveness and universality of this strategy in food traceability and authentication applications, five supramolecular fluorescence sensor arrays were designed as examples to study the detection of five foods from different origins or grades (summarized in **Table** **1**).

**Table 1:** Summary of the supramolecular fluorescence sensor arrays.

Sensor array	Sensor unit	Application in food
sensor array 1	Sensor unit 1 (SU 1): PyBTA + PSS	Apple traceability
	Sensor unit 2 (SU 2): PyBTA + SDS	
	Sensor unit 3 (SU 3): PyBTA + Sug	
sensor array 2	Sensor unit 4 (SU 4): PDI‐BTMA + PSS	Citrus traceability
	Sensor unit 5 (SU 5): PDI‐BTMA + SDS	
	Sensor unit 6 (SU 6): PDI‐BTMA + Sug	
sensor array 3	Sensor unit 3 (SU 3): PyBTA + Sug	Tea authentication
	Sensor unit 7 (SU 7): PyBA + Sug	
	Sensor unit 8 (SU 8): PyB + Sug	
sensor array 4	Sensor unit 5 (SU 5): PDI‐BTMA + SDS	Honey authentication
	Sensor unit 9 (SU 9): PDI‐HTMA + SDS	
	Sensor unit 10 (SU 10): PDI‐OTMA + SDS	
sensor array 5	Sensor unit 3 (SU 3): PyBTA + Sug in 0% DMSO	Coffee bean traceability
	Sensor unit 11 (SU 11): PyBTA + Sug in 20% DMSO	
	Sensor unit 12 (SU 12): PyBTA + Sug in 40% DMSO	

### 2.2 Application in Apple Traceability

**Figure 4:**
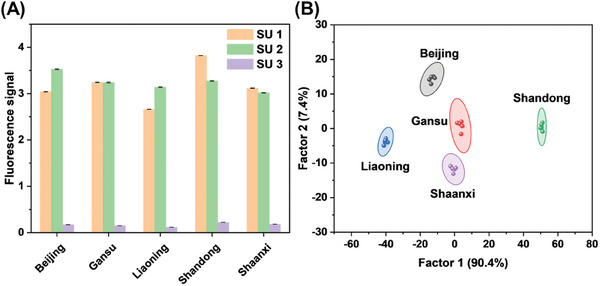
A) Fluorescence response patterns of sensor array 1 to apple samples from five regions. B) LDA score plots for the discrimination of apple samples from five regions with 95% confidence ellipses.

Apple is one of the most popular fruits consumed on a global scale, and China holds a dominant position in its production. Due to differences in environment, soil characteristics, and other factors, notable distinctions arise in the apple quality among different regions. Apple traceability can provide technical support for the protection of geographical indication products.^[^ ^27^ ^]^ Sensor array 1, consisting of supramolecular assemblies of a pyrene derivative and various cofactors (mentioned above), was first tried to be applied to distinguish apples from different origins. Figure S9 (Supporting Information) illustrated that PSS and SDS could induce the aggregation of PyBTA to generate an excimer emission band^[^ ^28^ ^]^; whereas Sug could encapsulate PyBTA to form a host–guest complex, thereby changing the microenvironment of PyBTA to increase its monomer emission (375–420 nm).^[^ ^29^ ^]^ It was noted that interactions in sensor array 1 could be converted into fluorescence signals. Upon addition of apple samples, SU 1–3 exhibited different fluorescence responses, which could be attributed to the disruption of three supramolecular assemblies (Figure S10, Supporting Information). Meanwhile, one sensor unit also gave different information about apple samples from five regions in China (**Figure** **4**A). It is indicated that three sensor units presented unique response patterns for each apple sample. Through these differentiated fluorescence responses, apples from different origins could be distinguished by linear discriminant analysis (LDA). The two most significant LDA factors (F1  =  90.4% and F2  =  7.4%) were used to obtain a 2D score plot with 95% confidence ellipses, and all 25 points (5 apple samples × 5 replicates) were well clustered into five distinct groups without any overlap (Figure 4B, original data for LDA is in Table S2, Supporting Information). To further test the ability of sensor array 1 to identify unknown samples, ten unknown apple samples from various regions were measured and analyzed with the same training matrix by LDA. The results in Figure S11 (Supporting Information) showed all unknown samples were correctly classified, indicating that this model based on sensor array 1 could be successfully used to trace the origin of apples.

### 2.3 Application in Citrus Traceability

**Figure 5:**
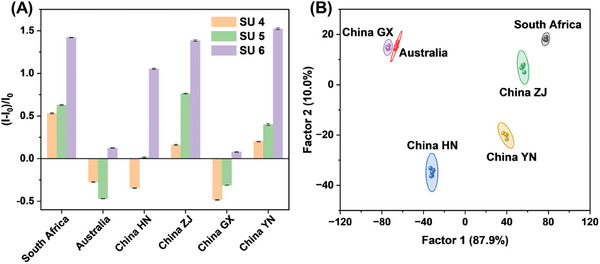
A) Fluorescence response patterns of sensor array 2 to citrus samples from six regions. B) LDA score plots for the discrimination of citrus samples from six regions with 95% confidence ellipses. HN, Hunan Province; ZJ, Zhejiang Province; GX, Guangxi Province; YN, Yunnan Province.

The second application was aimed at citrus traceability that provided brand protection for premium citrus origins. Sensor array 2 was constructed by PDI‐BTMA and three cofactors through non‐covalent interactions, including PDI‐BTMA/PSS (SU 4), PDI‐BTMA/SDS (SU 5), and PDI‐BTMA/Sug (SU 6). Figure S12 (Supporting Information) illustrated that the formation of supramolecular assemblies of PDI‐BTMA with cofactors changed its fluorescence. Due to the aggregation causing quenching (ACQ) effect of perylene derivatives,^[^ ^30^ ^]^ a “turn‐off” fluorescence state of SU 4–6 proved that cofactors caused PDI‐BTMA to aggregate. When citrus samples were added, they would interfere with interactions in three supramolecular assemblies and change the fluorescence signals of the sensing units (Figure S13, Supporting Information). As illustrated in **Figure** **5**, sensor array 2 gave a distinct recognition pattern for each citrus sample, and clusters representing citrus samples from six different origins were located in different areas according to LDA (original data for LDA is in Table S3, Supporting Information). For ten unknown citrus samples, the classification accuracy of this approach was 100% (Figure S14, Supporting Information), revealing good discrimination performance of sensor array 2.

### 2.4 Application in Tea Authentication

**Figure 6:**
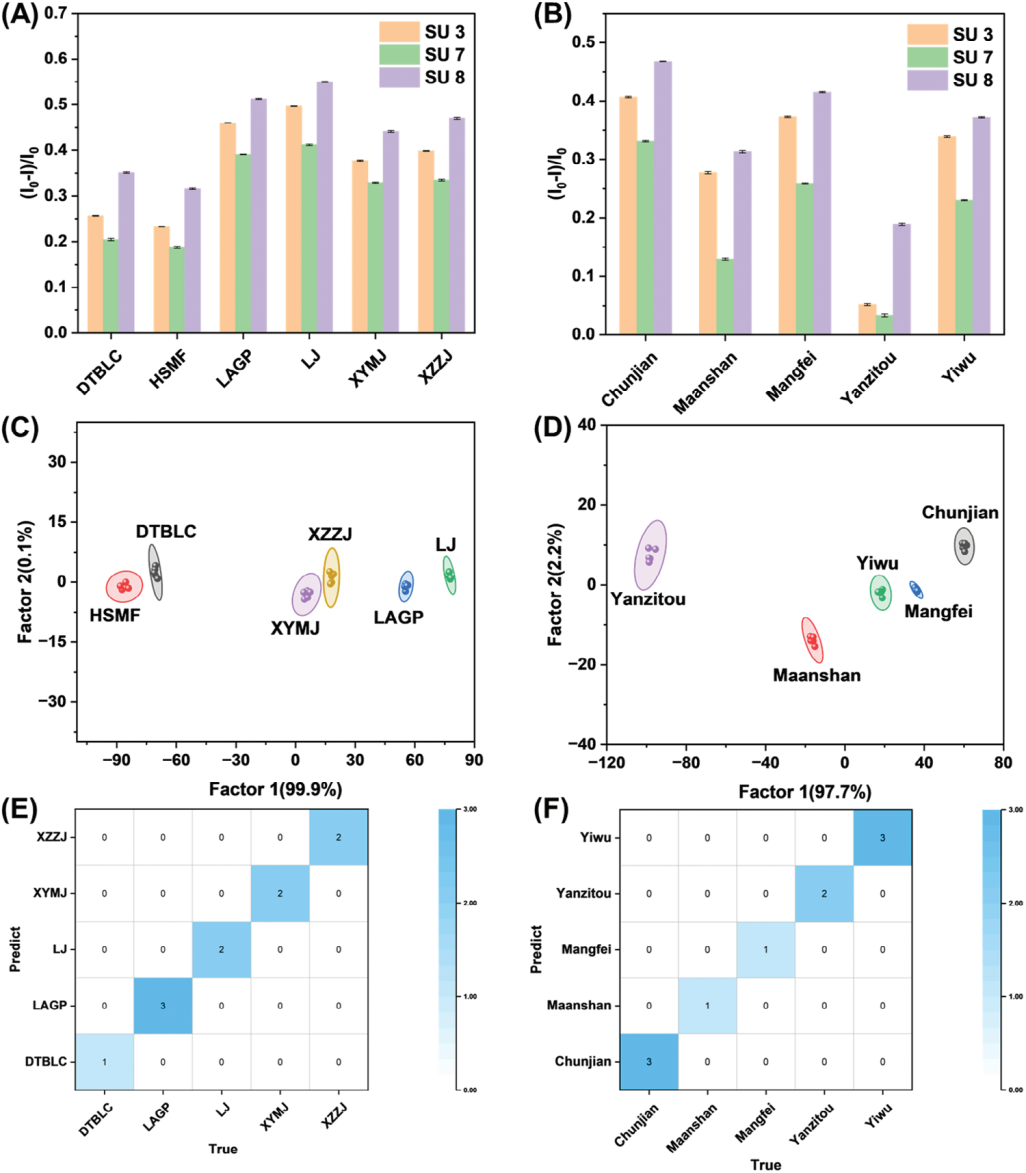
Fluorescence response patterns of sensor array 3 to A) six types of green tea and B) five types of Pu‐erh tea. LDA score plots for the discrimination of C) six types of green tea and D) five types of Pu‐erh tea with 95% confidence ellipses. Confusion matrix heatmap for classification predictions of E) green tea and F) Pu‐erh tea. DTBLC, Dongting Biluochun; HSMF, Huangshan Maofeng; LAGP, Lu an Guapian; LJ, Longjing Tea; XYMJ, Xinyang Maojian; XZZJ, Xianzhi Zhujian.

Tea classification could contribute to combatting counterfeit and low‐quality tea products and promote the development of a sustainable tea market. Sensor array 3, composed of PyBTA/Sug (SU 3), PyBA/Sug (SU 7), and PyB/Sug (SU 8) supramolecular assemblies, had been developed for tea identification. This sensor array introduced another feasible scheme for constructing sensor units by utilizing a variety of fluorescent probes, rather than a diversity of cofactors. The assembly of Sug with PyBTA/PyBA/PyB could enhance the monomer fluorescence emission of pyrene derivatives (Figure S15, Supporting Information), while the addition of tea samples could affect their host‐guest interactions to change the fluorescence intensity of sensing units (Figure S16, Supporting Information). It indicated that interactions between sensor array 3 and tea samples could be output through fluorescence signals. The results in **Figure** **6**A, C demonstrated that sensor array 3 had different responses to various types of green tea samples and could achieve 100% accurate discrimination of six types of green tea through LDA (original data for LDA is in Table S4, Supporting Information). In the classification of Pu‐erh tea, a kind of dark tea, in five different grades, sensor array 3 exhibited great performance as well (Figure 6B,D, original data for LDA is in Table S5, Supporting Information). Moreover, Figure 6E,F showed that all unknown green tea and Pu‐erh tea samples could be predicted and identified correctly by the above models.

### 2.5 Application in Honey Authentication

**Figure 7:**
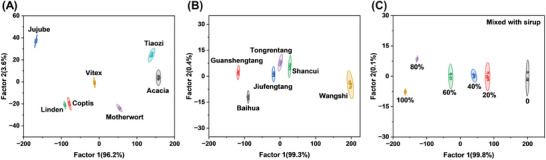
LDA score plots for the discrimination of A) seven types of honey from Wang's brand, B) six brands of acacia honey, and C) honey adulterated with different proportions of sirup.

Honey from different botanical and geographical origins differ significantly in their market value due to their quality, flavor, or health benefits. In sensor array 4, SDS was assembled with PDI‐BTMA, PDI‐HTMA, and PDI‐OTMA respectively to form SU 5, SU9, and SU 10, trying to authenticate different types and grades of honey. Similar to PDI‐BTMA, SDS could also induce the fluorescence quenching of PDI‐HTMA and PDI‐OTMA (Figure S17, Supporting Information). The addition of honey samples could damage the interactions between SDS and PDI‐BTMA/PDI‐HTMA/PDI‐OTMA in different degrees to make sensor array 4 output fluorescence signals (Figure S18, Supporting Information). Due to the unique molecular interactions between different honey samples and the supramolecular assemblies in sensor array 4, seven different types of honey of the same brand, six different brands of honey of the same type, and honey adulterated with different proportions of sirup would have distinctive fluorescence signal patterns (Figure S19, Supporting Information) and could be correctly classified by applying LDA to these patterns (**Figure** **7**, original data for LDA is in Tables S6–S8, Supporting Information). It could be seen from the results in Figure S20 (Supporting Information) that the prediction accuracy of these classification models for unknown honey sample types, brands, and adulteration levels was 100%. These findings proved that sensor array 4 could identify small differences in honey samples, providing a sensitive detection method for the quality control of honey products.

### 2.6 Application in Coffee Bean Traceability

**Figure 8:**
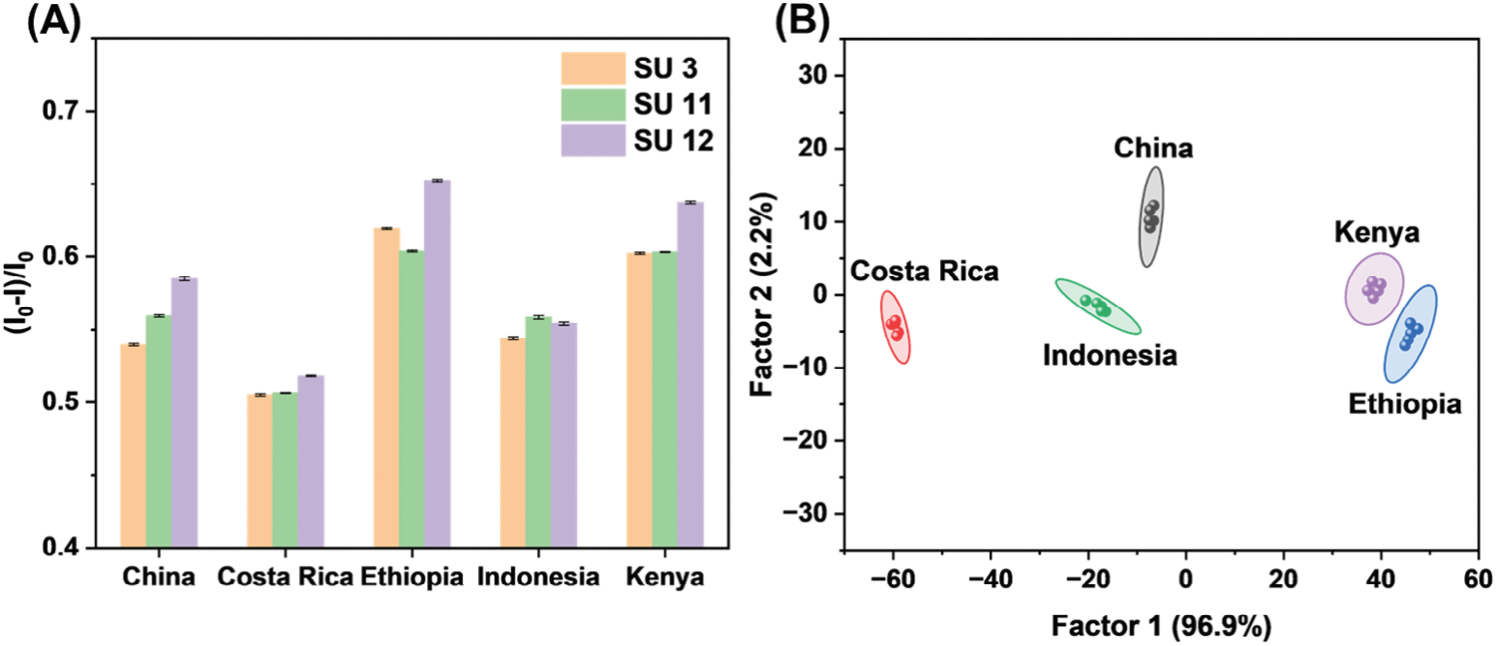
A) Fluorescence response patterns of sensor array 5 to coffee samples from five regions. B) LDA score plots for the discrimination of coffee samples from five regions with 95% confidence ellipses.

It is well known that the origins of coffee beans are closely related to their quality and price, so the development of coffee bean traceability methods can effectively prevent coffee bean fraud.^[^ ^31^ ^]^ We utilized PyBTA‐Sug supramolecular assembly in solvents containing 0% DMSO (SU 3), 20% (SU 11), and 40% DMSO (SU 12) to build sensor array 5 in an attempt to trace the origins of coffee beans. The interactions of supramolecular assemblies are influenced by the solvent microenvironment,^[^ ^32^ ^]^ so developing supramolecular fluorescence sensor units based on different solvents represents another viable scheme. Figure S21 (Supporting Information) showed that in solvents with different proportions of DMSO, the fluorescence intensities of PyBTA before and after being assembled with Sug were different. It meant that fluorescence signals could reflect the assembly state of PyBTA and Sug. Therefore, the changes in fluorescence (Figure S22, Supporting Information) illustrated that coffee bean samples could affect the interactions of three sensor units. The differences in coffee beans from different origins could be translated into specific fluorescence fingerprints of sensor array 5 (**Figure** **8**A). When the fluorescence fingerprints were analyzed by LDA, coffee beans from five various origins were well separated on the LDA plot (Figure 8B, original data for LDA is in Table S9, Supporting Information), and unknown coffee bean samples were identified with 100% classification accuracy (Figure S23, Supporting Information). It could be said that sensor array 5 was sensitive and effective for coffee bean traceability.

## 3 Conclusion

In conclusion, we have proposed a novel strategy for general food traceability and authentication based on supramolecular assembly sensor arrays regulated by non‐covalent interactions. The stimuli‐responsive and tunable nature of self‐assembly and the ubiquity of non‐covalent interactions endow this strategy with simple preparation, infinite diversity, rapid response, high sensitivity, and resolution. The sensing mechanism could be attributed to the assembly and disassembly between different sensor units and food molecules driven by electrostatic, hydrophobic, host–guest, π─π interactions, etc. As a proof of concept, five fluorescence sensor arrays based on perylene/pyrene derivatives assemblies were designed and successfully applied to the traceability or authentication of apples, citrus, tea, honey, and coffee beans. An accuracy of 100% was achieved for each unknown sample. We believe that similar assemblies based on this strategy would not only shed new light on traceability and authentication of various foods but also provide valuable insights for the construction of a general sensing method.

## Conflict of Interest

The authors declare no conflict of interest.

## Supporting information

Supporting Information

## Data Availability

The data that support the findings of this study are available from the corresponding author upon reasonable request.
