# Supplementary material for: A General Strategy for Food Traceability and Authentication Based on Assembly‐Tunable Fluorescence Sensor Arrays
Source: Adv Sci (Weinh). 2024 May 17;11(28):2309259. doi: 10.1002/advs.202309259 (PMC11267353; doi:10.1002/advs.202309259)
Supplement: Supplementary file 1 — Supporting Information [file ADVS-11-2309259-s001.docx]

Supporting Information

A general strategy for food traceability and authentication based on assembly-tunable fluorescence sensor arrays

He Cheng^†^, Tianyue Liu^†^, Jingsheng Tian, Ruixuan An, Yao Shen, Mingxi Liu, Zhiyi Yao*

Beijing Laboratory of Food Quality and Safety, College of Food Science and Nutritional Engineering, China Agricultural University, Beijing 100083, China

*Corresponding author: E-mail: yaozy@cau.edu.cn. Tel: +86-10-62737055; Fax: +86-10-62737055.

**Experimental section**

**Materials**

Poly (sodium 4-styrenesulfonate) with an average molecular weight of 70000 was purchased from Alfa Aesar Co., Ltd. Sugammadex sodium was purchased from Chengdu Novel Biochemical Technology Co., Ltd. Perylene-3, 4, 9, 10-tetracarboxylic dianhydride was obtained from Hubei Xinmingtai Chemical Co., Ltd. D_2_O and deuterated trifluoroacetic acid were purchased from Beijing Innochem Science & Technology Co., Ltd. 1-pyrenebutyric acid was purchased from J&K Chemical Co., Ltd. 1-pyrenebutanol was purchased from Accela ChemBio Co., Ltd. Sodium alginate was purchased from Beijing Solarbio Science & Technology Co., Ltd. All other reagents and solvents were purchased from Shanghai Macklin Biochemical Co., Ltd., Shanghai Aladdin Bio-Chem Technology Co., Ltd. and Beijing Chemical Works Co., Ltd. These above-mentioned reagents and solvents were used as received. Apple and citrus samples were obtained from the supermarket in Haidian District, Beijing, China. Green tea, Pu-erh tea, honey, and coffee bean samples were all available brands in the Chinese market. The details of these food samples are shown in Table S1. All solutions were prepared using purified water from Hangzhou Wahaha Group Co., Ltd.

**Table S1.** Detailed information on various food samples.

| **Food Sample** | | **Details** |
| --- | --- | --- |
| Apple | Sample 1 | **Category**: Red Fuji Apple; **Origin**: Changping, Beijing, China |
|  | Sample 2 | **Category**: Red Fuji Apple; **Origin**: Pingliang, Gansu, China |
|  | Sample 3 | **Category**: Red Fuji Apple; **Origin**: Dalian, Liaoning, China |
|  | Sample 4 | **Category**: Qixia Fuji Apple; **Origin**: Yantai, Shandong, China |
|  | Sample 5 | **Category**: Red Fuji Apple; **Origin**: Yan'an, Shaanxi, China |
| Citrus | Sample 1 | **Category**: Guangxi Clementine; **Origin**: Wuzhou, Guangxi, China |
|  | Sample 2 | **Category**: Yongquan Tangerine; **Origin**: Taizhou, Zhejiang, China |
|  | Sample 3 | **Category**: Dongjiang Lake Tangerine; **Origin**: Zixing, Hunan, China |
|  | Sample 4 | **Category**: Huaning Citrus; **Origin**: Yuxi, Yunnan, China |
|  | Sample 5 | **Category**: Satsumas; **Origin**: South Africa |
|  | Sample 6 | **Category**: Afourer; **Origin**: Australian |
| Green tea | Sample 1 | **Category**: Dongting Biluochun  **Origin**: Suzhou Sanwanchang Tea Co., Ltd. Suzhou, Jiangsu, China  **Brand**: Beijing Zhangyiyuan Tea Co., Ltd. |
|  | Sample 2 | **Category**: Huangshan Maofeng  **Origin**: Beijing Zhangyiyuan Jingtailong Tea Co., Ltd. Huangshan, Anhui, China  **Brand**: Beijing Zhangyiyuan Tea Co., Ltd. |
|  | Sample 3 | **Category**: Lu an Guapian  **Origin**: Jinzhai County Qifu Organic Tea Factory, Lu'an, Anhui, China  **Brand**: Beijing Zhangyiyuan Tea Co., Ltd. |
|  | Sample 4 | **Category**: Qiantang Longjing  **Origin**: Zhangjiashan Tea Farm, Zhongtai Township, Yuhang District, Hangzhou, Zhejiang, China  **Brand**: Beijing Zhangyiyuan Tea Co., Ltd. |
|  | Sample 5 | **Category**: Xinyang Maojian  **Origin**: Huangchuan County Guangzhou Tea Co., Ltd. Xinyang, Henan, China  **Brand**: Beijing Zhangyiyuan Tea Co., Ltd. |
|  | Sample 6 | **Category**: Xianzhi Zhujian  **Origin**: Beijing Zhangyiyuan Jinqiao Tea Co., Ltd. Leshan, Sichuan, China  **Brand**: Beijing Zhangyiyuan Tea Co., Ltd. |
| Pu-erh tea | Sample 1 | **Brand**: Yunnan Shuangjiang Mengku Tea Co., Ltd.  **Series**: Chunjian; **Origin**: Lincang Tea Area, Yunnan, China |
|  | Sample 2 | **Brand**: Yunnan Rongshi Yongde Tea Co., Ltd.  **Series**: Maanshan; **Origin**: Lincang Tea Area, Yunnan, China |
|  | Sample 3 | **Brand**: Yunnan Rongshi Yongde Tea Co., Ltd.  **Series**: Mangfei; **Origin**: Lincang Tea Area, Yunnan, China |
|  | Sample 4 | **Brand**: Yunnan Rongshi Yongde Tea Co., Ltd.  **Series**: Yanzitou; **Origin**: Lincang Tea Area, Yunnan, China |
|  | Sample 5 | **Brand**: Yunnan Shuangjiang Mengku Tea Co., Ltd.  **Series**: Yiwu; **Origin**: Lincang Tea Area, Yunnan, China |
| Honey | Sample 1 | **Category**: Acacia honey; **Brand**: Wangshi |
|  | Sample 2 | **Category**: Coptis honey; **Brand**: Wangshi |
|  | Sample 3 | **Category**: Jujube honey; **Brand**: Wangshi |
|  | Sample 4 | **Category**: Linden honey; **Brand**: Wangshi |
|  | Sample 5 | **Category**: Motherwort honey; **Brand**: Wangshi |
|  | Sample 6 | **Category**: Vitex honey; **Brand**: Wangshi |
|  | Sample 7 | **Category**: Tiaozi honey; **Brand**: Wangshi |
|  | Sample 8 | **Category**: Acacia honey; **Brand**: Guanshengyuan |
|  | Sample 9 | **Category**: Acacia honey; **Brand**: Jiufengtang |
|  | Sample 10 | **Category**: Acacia honey; **Brand**: Shancui, China Oil & Foodstuffs Corporation |
|  | Sample 11 | **Category**: Acacia honey; **Brand**: Baihua |
|  | Sample 12 | **Category**: Acacia honey; **Brand**: Tongrentang |
| Sirup | | **Category**: Golden sirup; **Brand**: Taikoo |
| Coffee bean | Sample 1 | **Brand**: SINLOY; **Origin**: Yunnan, China; **Category**: Yunnan arabica coffee |
|  | Sample 2 | **Brand**: SINLOY; **Origin**: Costa Rica |
|  | Sample 3 | **Brand**: SINLOY; **Origin**: Ethiopia; **Category**: Oiran coffee beans |
|  | Sample 4 | **Brand**: SINLOY; **Origin**: Indonesia; **Category**: Mandheling coffee |
|  | Sample 5 | **Brand**: SINLOY; **Origin**: Kenya; **Category**: SL34 |

**Instruments**

The fluorescence data were recorded by the FluoroMax spectrophotometer (HORIBA Scientific, Japan). ^1^H-NMR spectra were obtained from a Bruker Ascend 500 NMR spectrometer (Bruker, Germany). The transmission electron microscopy (TEM) images were obtained with a JEM-1200EX transmission electron microscope (JEOL Ltd, Japan). The pH values were measured by a PHS-25 pH meter (INESA Scientific Instrument, China). Food samples were centrifuged through an MS-12Pro centrifuge (JOANLAB Equipment CO., Ltd, China).

**Synthesis and characterization of fluorescent probe**

**PyBTA** was synthesized and purified according to our previous work^[1]^.


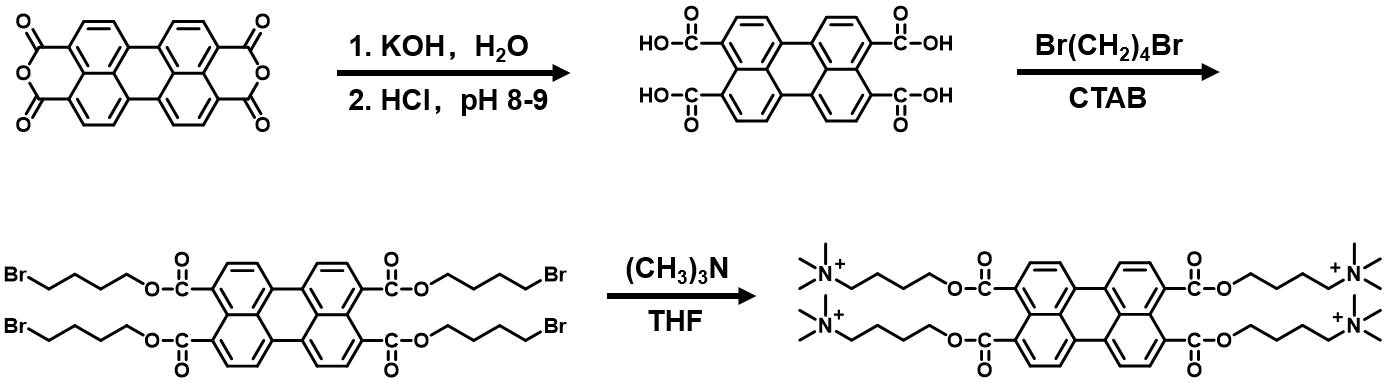


**Scheme S1.** The synthesis procedures of PDI-BTMA.

**Synthesis of PDI-BTMA**^[2]^**:** Perylene-3,4,9,10-tetracarboxylic dianhydride (784 mg, 2.0 mmol), KOH (1.0 g, 17.7 mmol), and 30 mL deionized water was added into a 100 mL flask and stirred at 80 °C for 30 min. The solution was filtered and its pH value was adjusted to 8-9 with 2 M HCl. Subsequently, cetyltrimethylammonium bromide (CTAB) (400 mg, 1.1 mmol) was added. The mixture was stirred vigorously for 10 min, and 1,4-dibromobutane (4.3 g, 20.0 mmol) was added. The solution was refluxed with vigorous stirring at 125 °C for 3 h. The aqueous solution became clear and colorless, and on top of it, a layer of red oil formed. Next, CHCl_3_ (30 mL) was added to the reaction mixture. The organic phase was washed three times with 15 % aqueous NaCl (30 mL) and concentrated under reduced pressure. The crude product was purified by column chromatography on silica gel using a mixture of CH_2_Cl_2_/petroleum ether (1:1) as eluent. After drying under vacuum, 3,4,9,10-tetra-(4-bromobutyloxy-carbonyl)-perylene was obtained as an orange solid. 3,4,9,10-tetra-(4-bromobutyloxy-carbonyl)-perylene was dissolved in THF (50 mL) in a 100 mL flask and an excess amount of trimethylamine (5 mL, 30 % aqueous solution) was added. The solution was refluxed at 80 °C for 3 days. During this period, water was added at several intervals (a total of 15 mL). The organic solvent was evaporated under reduced pressure, and the aqueous solution was washed with CHCl_3_ (30 mL) three times. After solvent evaporation and drying under vacuum, PDI-BTMA was obtained as a red solid. ^1^H NMR (500 MHz, D_2_O) δ (ppm): 7.67 (d, 4H), 7.58 (s, 4H), 4.44 (t, 8H), 3.44 (t, 8H), 3.11 (s, 36H), 2.05-1.97 (m, 8H), 1.97-1.89 (m, 8H).


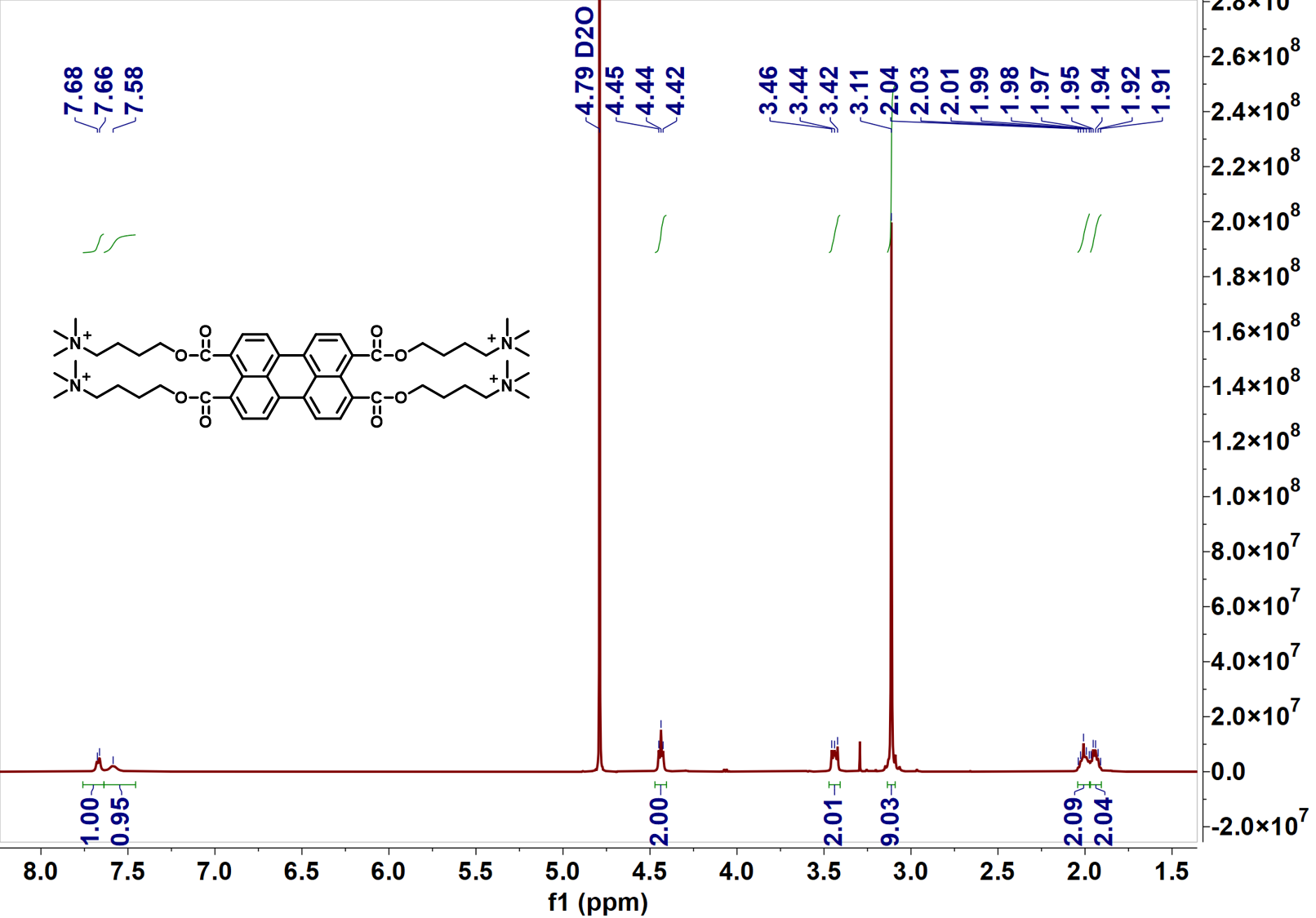


**Figure S1.** ^1^H NMR spectrum of PDI-BTMA.


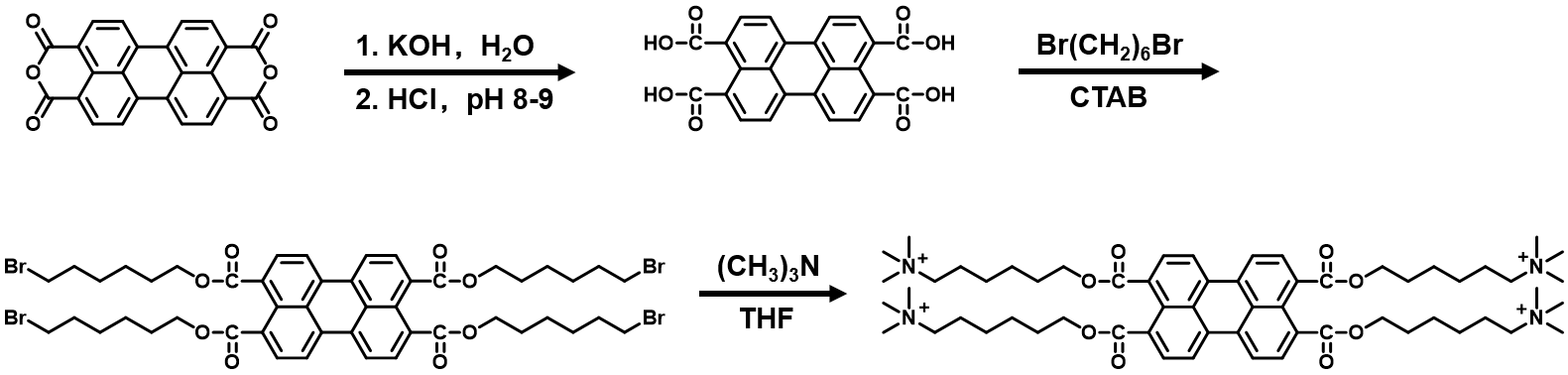


**Scheme S2.** The synthesis procedures of PDI-HTMA.

**Synthesis of PDI-HTMA:** The synthesis of PDI-HTMA was similar to that of PDI-BTMA except that the reactant 1,4-dibromobutane was replaced by 1,6-dibromohexane. ^1^H NMR (trifluoroacetic Acid-d, 500 MHz) δ (ppm): 8.63 (d, 4H), 8.27 (d, 4H), 4.61 (t, 8H), 3.48 (s, 8H), 3.26 (s, 36H), 2.02 (s, 16H), 1.74 (s, 8H), 1.64 (s, 8H).


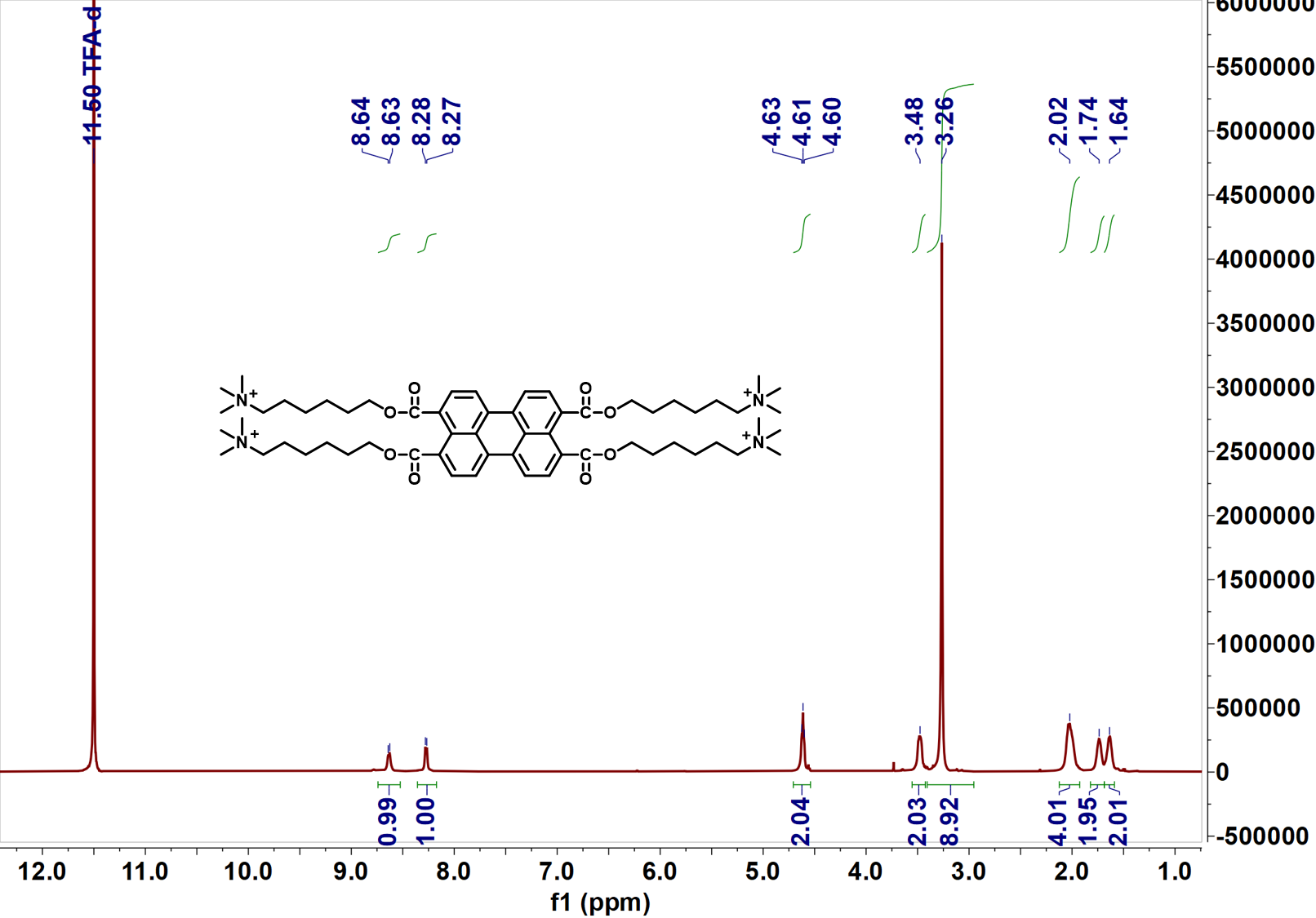


**Figure S2.** ^1^H NMR spectrum of PDI-HTMA.


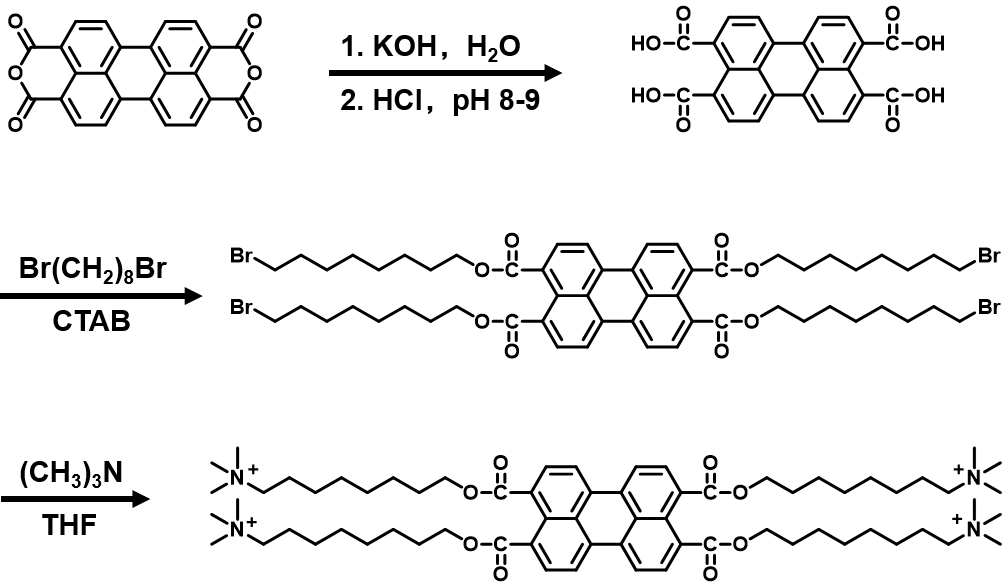


**Scheme S3.** The synthesis procedures of PDI-OTMA.

**Synthesis of PDI-OTMA:** The synthesis of PDI-OTMA was similar to that of PDI-BTMA except that the reactant 1,4-dibromobutane was replaced by 1, 8-dibromooctane. ^1^H NMR (trifluoroacetic Acid-d, 500 MHz) δ (ppm): 8.56 (s, 4H), 8.21 (s, 4H), 4.54 (t, 8H), 3.38 (s, 8H), 3.19 (s, 36H), 1.91 (d, 16H), 1.58 (s, 8H), 1.50 (s, 24H).


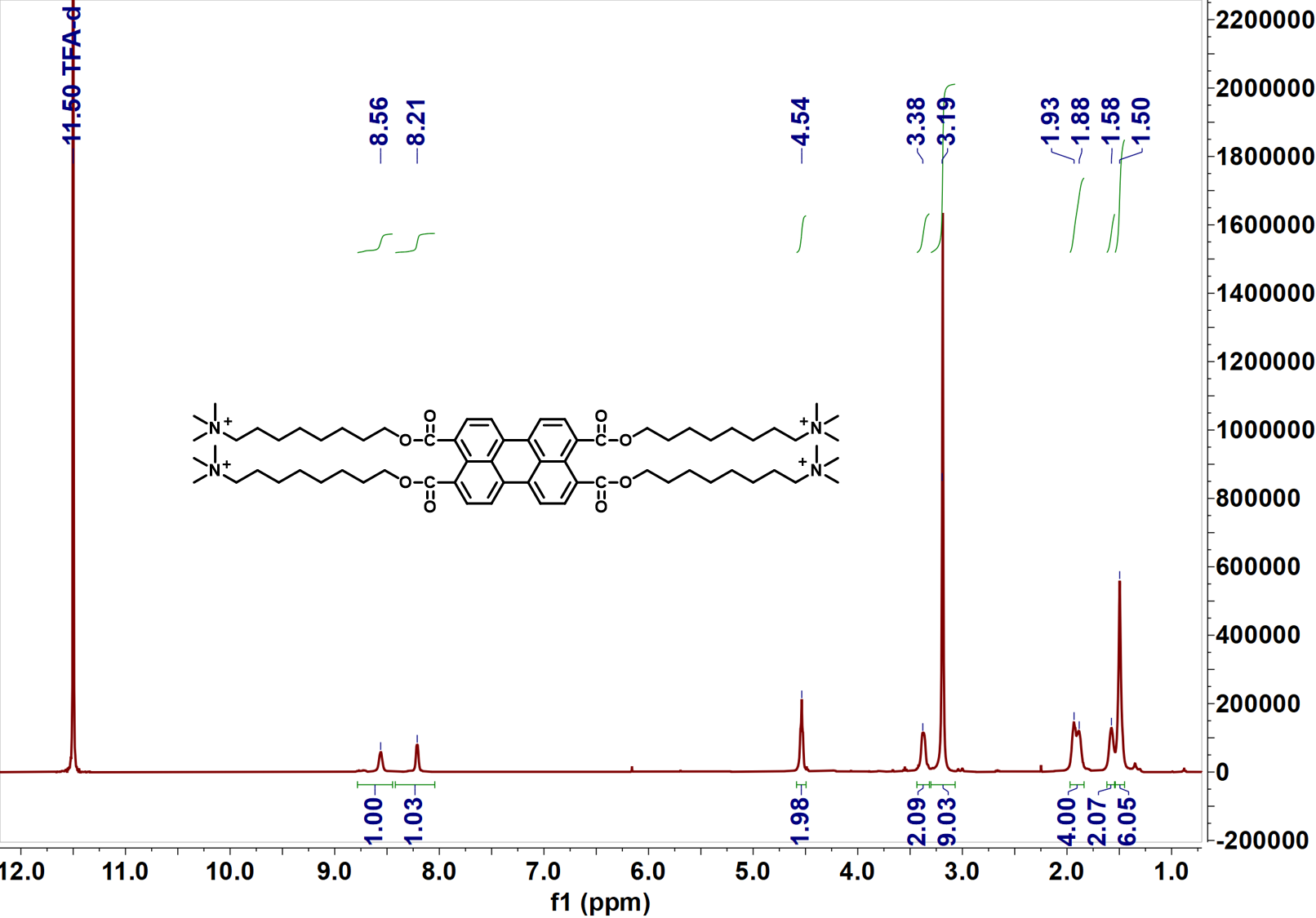


**Figure S3.** ^1^H NMR spectrum of PDI-OTMA.

**Sample preparation**

**Apple sample preparation:** The apples were first washed, cored, and stems removed, and then squeezed according to pulp/water 1:1 (mass ratio). The apple juice was centrifuged at 12000 rpm for 5 min, and the supernatant was passed through the 13 mm diameter sterile syringe filter with a 0.22 µm pore size hydrophilic PES membrane (Tianjin Jinteng Experimental Equipment Co., Ltd.) to obtain the apple sample. 50 μL apple sample was added to 1 mL HEPES buffer (10 mM, pH = 7) containing a sensing unit for fluorescence testing instantly.

**Citrus sample preparation:** The citrus was peeled and the pulp was squeezed directly. The citrus juice was centrifuged at 12000 rpm for 5 min, and the supernatant was passed through the same syringe filter as above to obtain the citrus sample. 50 μL citrus sample was added to 1 mL HEPES buffer (10 mM, pH = 7) containing a sensing unit for fluorescence testing instantly.

**Tea sample preparation:** After soaking 3 g tea in 150 mL boiling water for 5 min, the tea leaves were filtered out, and the tea soup was passed through the same syringe filter as above to obtain the tea sample. 50 μL tea sample was added to 1 mL HEPES buffer (10 mM, pH = 7) containing a sensing unit for fluorescence testing instantly.

**Honey sample preparation:** Dissolve the honey according to honey/water 1:5 (mass ratio), and then pass through the same syringe filter as above to obtain the honey sample. Specific concentrations of sensing units were directly added to 1 mL honey samples for fluorescence testing instantly.

**Coffee bean sample preparation:** Coffee beans were ground into coffee powder by the Mahlkönig VTA6S Shop Grinder. 1g coffee powder was soaked in 10mL boiling water for 5min, and filtered through filter paper to obtain the filtrate. The filtrate was passed through the same syringe filter as above to obtain the coffee bean sample. 5 μL coffee sample was added to 1 mL HEPES buffer containing a sensing unit for fluorescence testing instantly.

**Data Acquisition and Analysis**

The excitation wavelength of pyrene derivatives is 350 nm, and that of perylene derivatives is 440 nm. In **sensor array 1**, the concentration of PyBTA was 5 μM, and the concentrations of PSS, SDS, and Sug were 0.02 μM, 30 μM, and 10 μM, respectively. The fluorescence signals of sensor unit 1 and sensor unit 2 were the *I_485_/I_397_* values of their sensing system (*I_397_* and *I_485_* represent the fluorescence intensity at 397 and 485 nm, respectively), and the fluorescence signal of sensor unit 3 was *(I_0_-I)/I_0_* (the initial fluorescence intensity at 397 nm of sensor unit 3 were recorded as *I_0_*; the fluorescence intensity at 397 nm of sensing unit 3 after adding the analyte was *I*). In **sensor array 2**, the concentration of PDI-BTMA was 1 μM, and the concentrations of PSS, SDS, and Sug were 0.01 μM, 30 μM, and 1 μM, respectively. The fluorescence signals of **sensor array 2** were the *(I-I_0_)/I_0_* values at 490 nm. In **sensor array 3**, the concentration of PyBTA/PyBA/PyB was 5 μM, and the concentration of Sug was 10 μM. The fluorescence signals of **sensor array 3** were the same as that of sensor unit 3. In **sensor array 4**, the concentration of PDI-BTMA/PDI-HTMA/PDI-OTMA was 1 μM and the concentration of SDS was 30 μM. The fluorescence signals of **sensor array 4** were the same as that of **sensor array 2**. The concentrations and fluorescence signals of sensor units in **sensor array 5** were the same as those of sensor unit 3. Fluorescence measurements were taken immediately after each test sample was mixed well (about 10 s). Each experiment for array sensing analysis was repeated five times, and LDA was performed through IBM SPSS statistics 26.


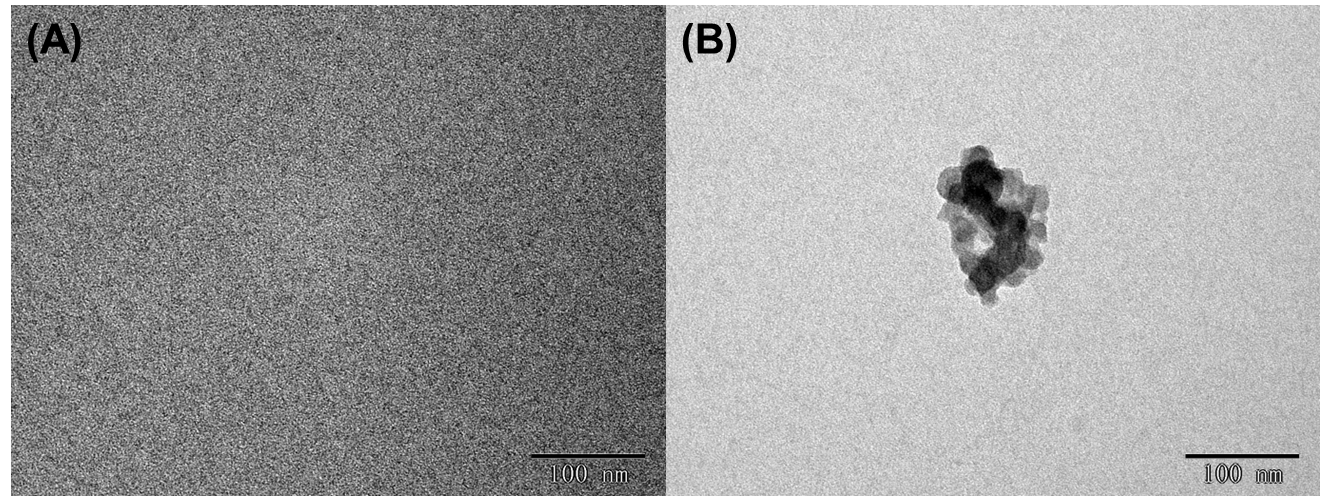


**Figure S4.** TEM images of (A) PyBTA (5 μM) and (B) assembly of PyBTA (5 μM) and SDS (30 μM), scale bar: 100 nm.


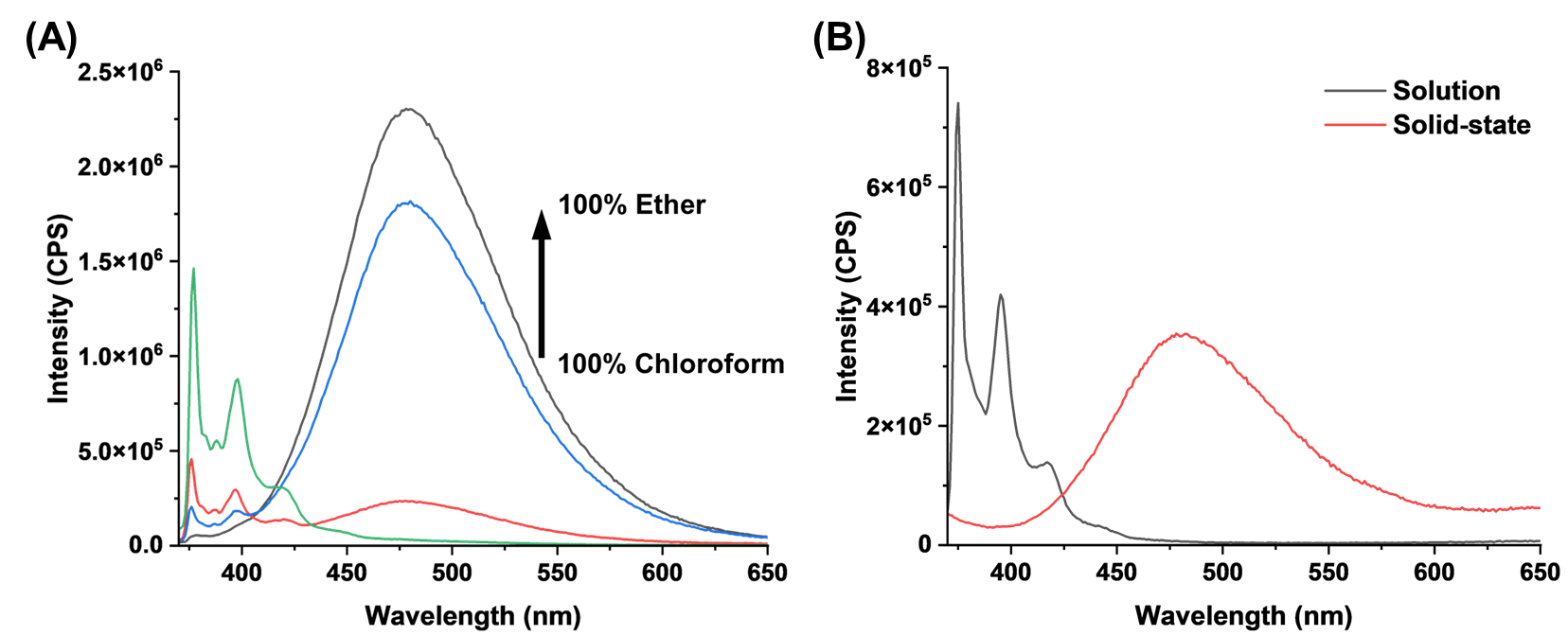


**Figure S5.** (A) Fluorescence spectra of PyBTA in different volume fractions of chloroform/ether, (B) fluorescence spectra of PyBTA solution and the solid-state PyBTA, λ_ex_ = 350 nm.


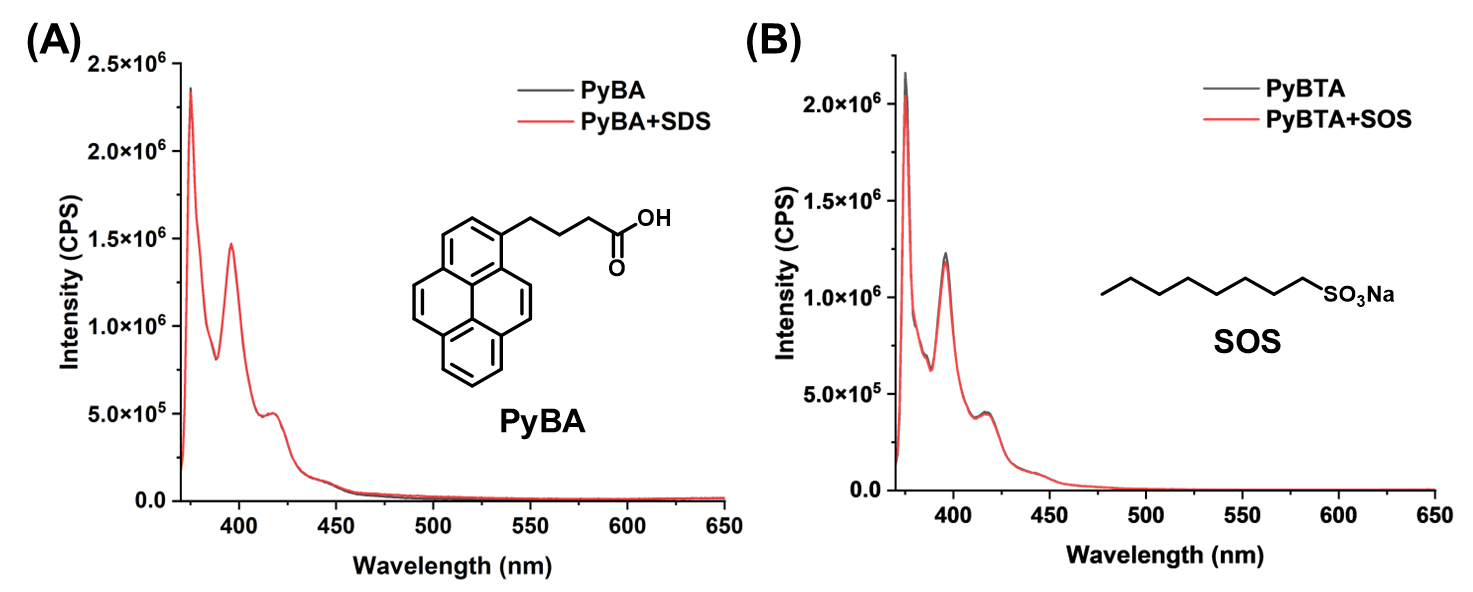


**Figure S6.** Fluorescence spectra of (A) PyBA (5 μM) and PyBA + SDS (30 μM), and (B) PyBTA (5 μM) and PyBTA + SOS (30 μM) in HEPES buffer (10 mM, pH = 7), *λ*_ex_ = 350 nm.


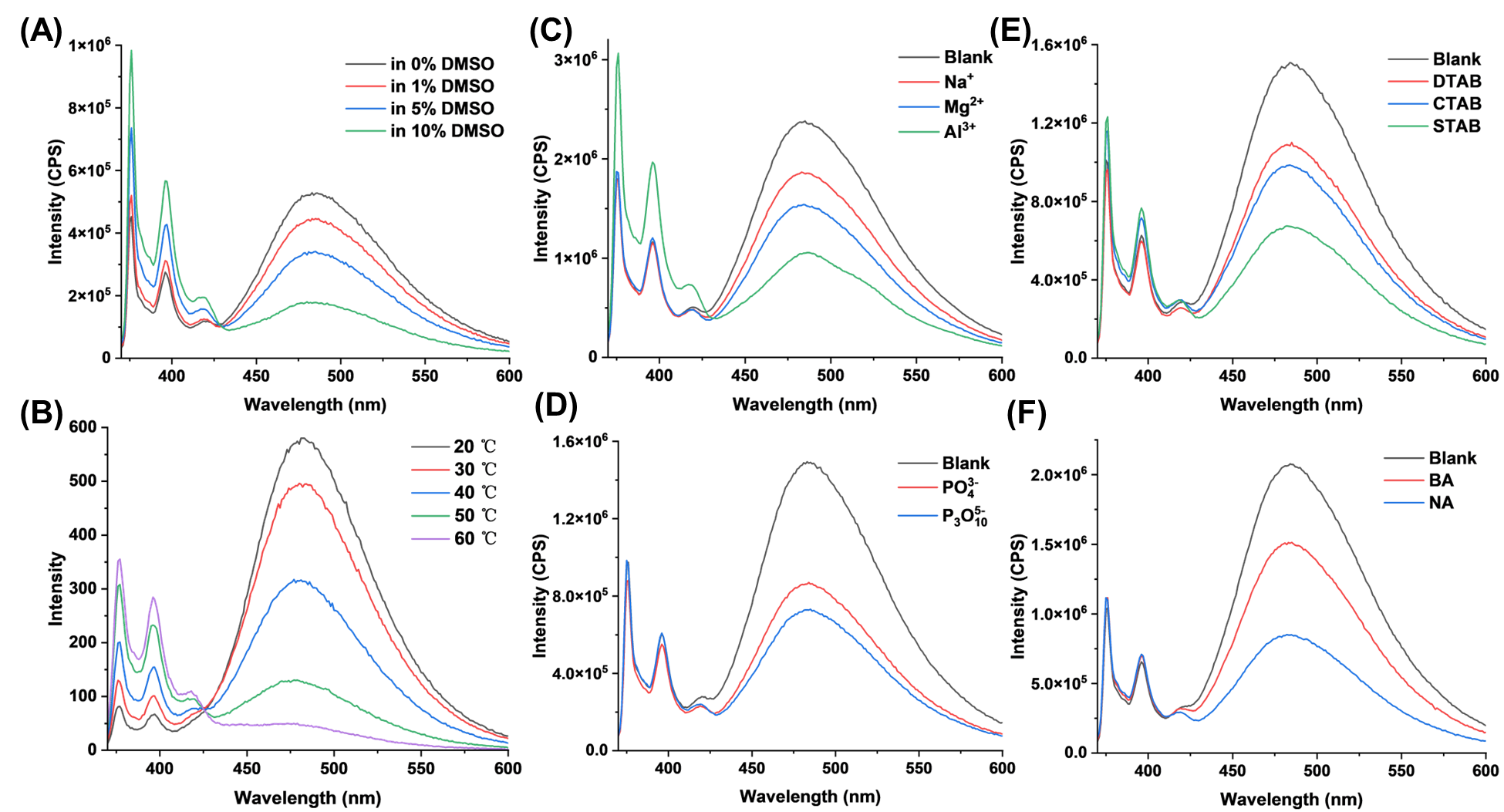


**Figure S7.** Fluorescence spectra of PyBTA-SDS (A) in different solvents, (B) under different temperatures, (C) with different cations, (D) with different anions, (E) with molecules of different carbon chain lengths, (F) with molecules of different aromatic ring structures. (DTAB: Dodecyltrimethylammonium bromide; CTAB: Cetyltrimethylammonium bromide; STAB: Stearyltrimethylammonium bromide; BA: Benzenesulfonic acid; NA: 2-Naphthalenesulfonic acid.)


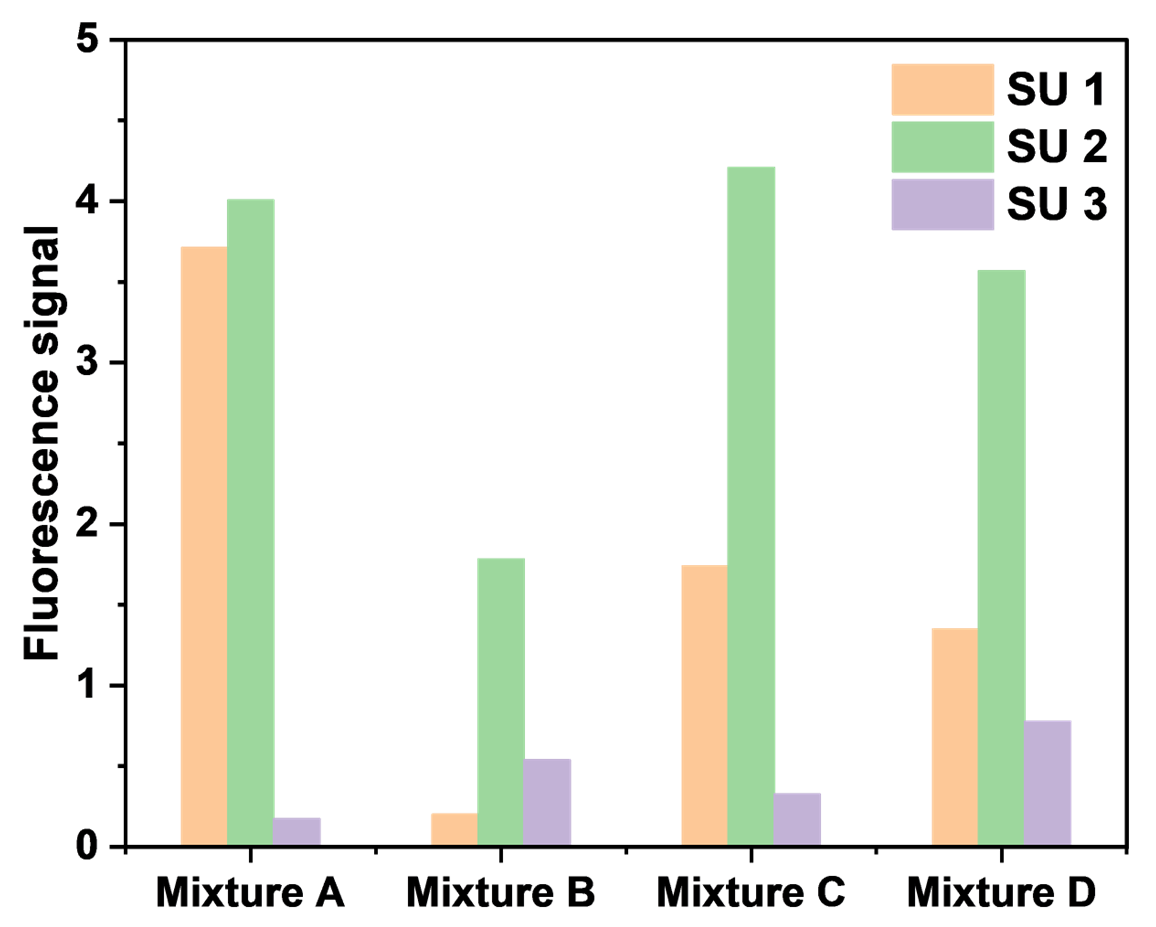


**Figure S8.** Fluorescence response patterns of **sensor array 1** to mixtures of various analytes in HEPES buffer (10 mM, pH = 7), Mixture A: Al^3+^ (0.1mM), CA (0.1mM), Caf (0.1mM), CGA (0.01mM), Fru (1mM), L-Arg (0.1mM), Suc (1mM), VB1 (0.1mM); Mixture B: Al^3+^ (0.5mM), CA (0.5mM), Caf (0.5mM), CGA (0.05mM), Fru (5mM), L-Arg (0.5mM), Suc (5mM), VB1 (0.5mM); Mixture C: Al^3+^ (0.1mM), CA (0.5mM), Caf (0.1mM), CGA (0.01mM), Fru (5mM), L-Arg (0.5mM), Suc (1mM), VB1 (0.5mM); Mixture D: Al^3+^ (0.5mM), CA (0.1mM), Caf (0.5mM), CGA (0.05mM), Fru (1mM), L-Arg (0.1mM), Suc (5mM), VB1 (0.1mM).


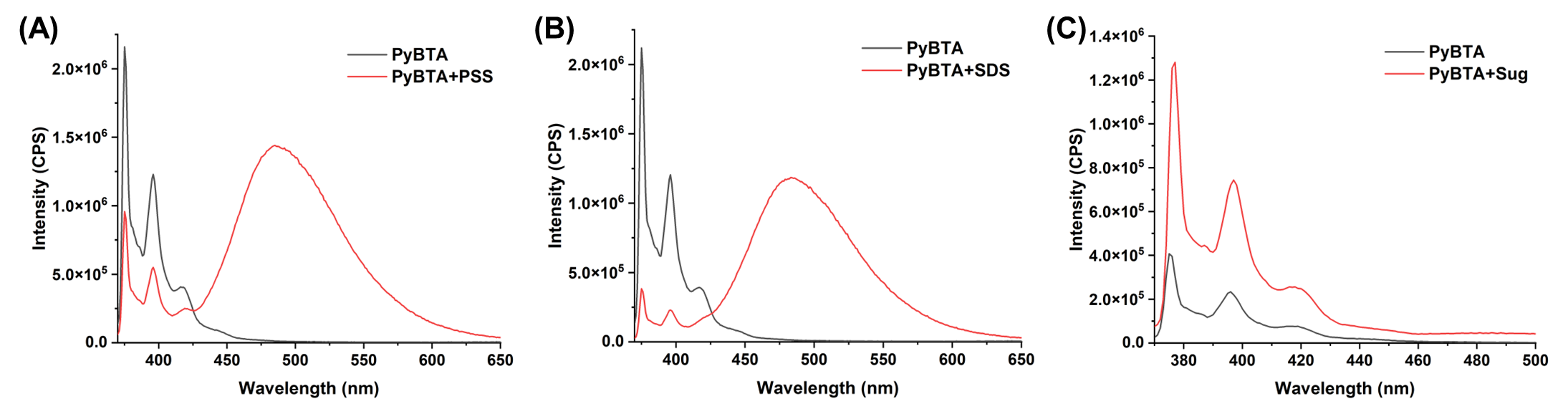


**Figure S9.** Fluorescence spectra of PyBTA (5 μM) before and after assembly with PSS (0.02 μM)/SDS (30 μM)/Sug (10 μM) in HEPES buffer (10 mM, pH = 7).


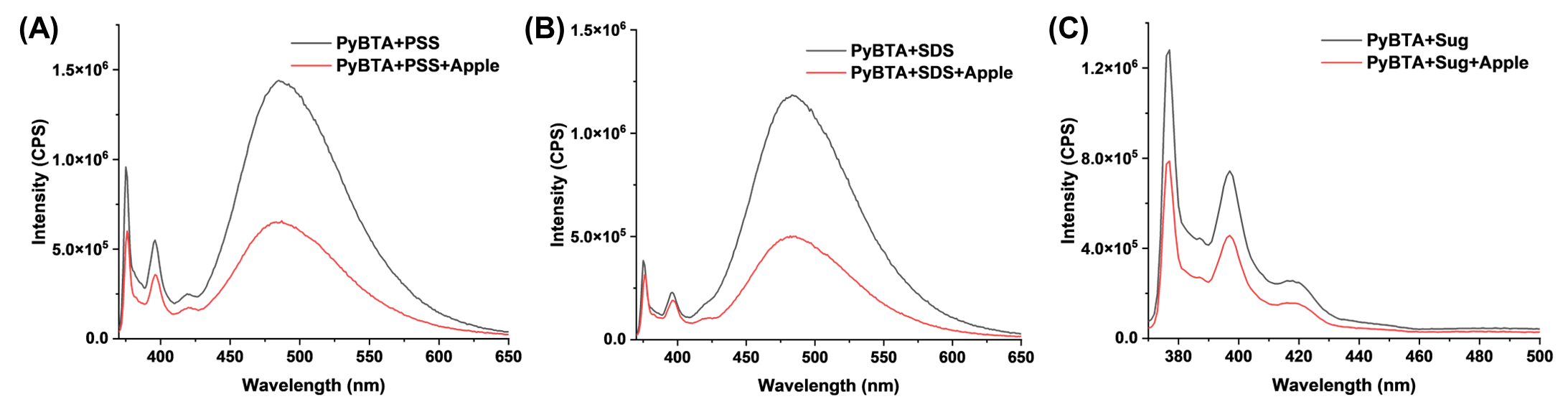


**Figure S10.** Fluorescence changes of sensor units in **sensor array 1** after the addition of apple sample in HEPES buffer (10 mM, pH = 7), *λ*_ex_ = 350 nm.

**Table S2.** The training matrix of fluorescence response patterns of **sensor array 1** against different apple samples.

|  | **SU 1** | **SU 2** | **SU 3** |
| --- | --- | --- | --- |
| Beijing | 3.0511 | 3.5429 | 0.1744 |
| Beijing | 3.0648 | 3.5316 | 0.1690 |
| Beijing | 3.0399 | 3.5371 | 0.1680 |
| Beijing | 3.0348 | 3.4937 | 0.1635 |
| Beijing | 3.0165 | 3.5257 | 0.1732 |
| Gansu | 3.2565 | 3.2614 | 0.1461 |
| Gansu | 3.2226 | 3.2558 | 0.1463 |
| Gansu | 3.2387 | 3.2531 | 0.1512 |
| Gansu | 3.2586 | 3.2371 | 0.1469 |
| Gansu | 3.2376 | 3.1955 | 0.1516 |
| Liaoning | 2.6586 | 3.1409 | 0.1169 |
| Liaoning | 2.6685 | 3.1588 | 0.1166 |
| Liaoning | 2.6758 | 3.1395 | 0.1206 |
| Liaoning | 2.6612 | 3.1477 | 0.1172 |
| Liaoning | 2.6504 | 3.1157 | 0.1123 |
| Shandong | 3.8208 | 3.2470 | 0.2252 |
| Shandong | 3.8228 | 3.3015 | 0.2305 |
| Shandong | 3.8178 | 3.2684 | 0.2231 |
| Shandong | 3.8103 | 3.2659 | 0.2227 |
| Shandong | 3.8231 | 3.2835 | 0.2204 |
| Shaanxi | 3.0995 | 3.0338 | 0.1796 |
| Shaanxi | 3.1290 | 3.0171 | 0.1803 |
| Shaanxi | 3.1135 | 3.0184 | 0.1791 |
| Shaanxi | 3.1356 | 3.0254 | 0.1832 |
| Shaanxi | 3.1100 | 2.9932 | 0.1840 |


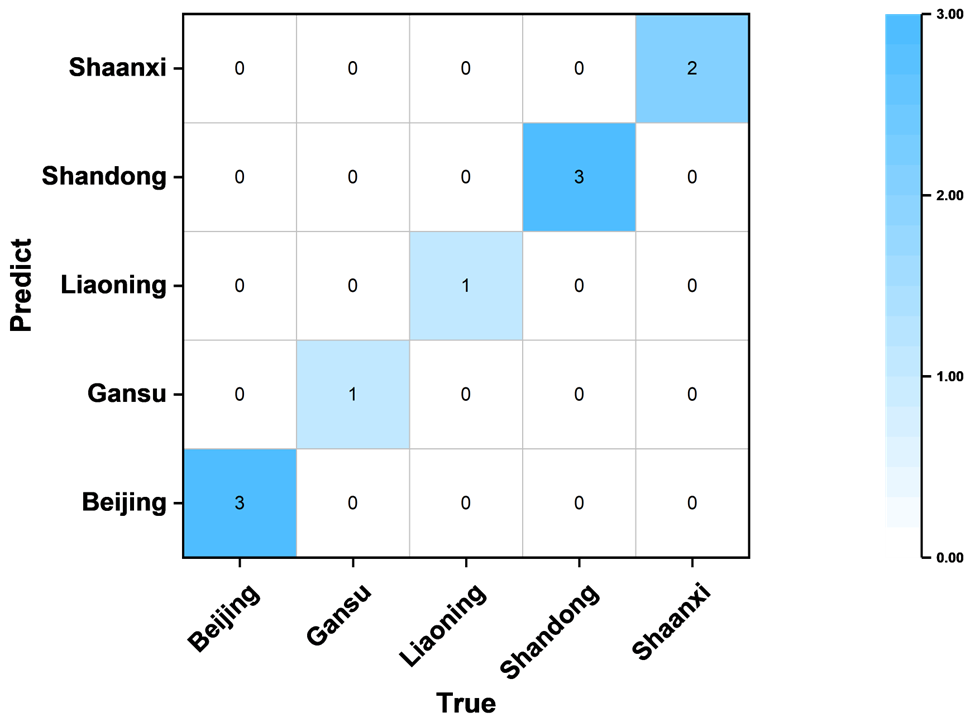


**Figure S11.** Confusion matrix heatmap for classification predictions of apple regions.


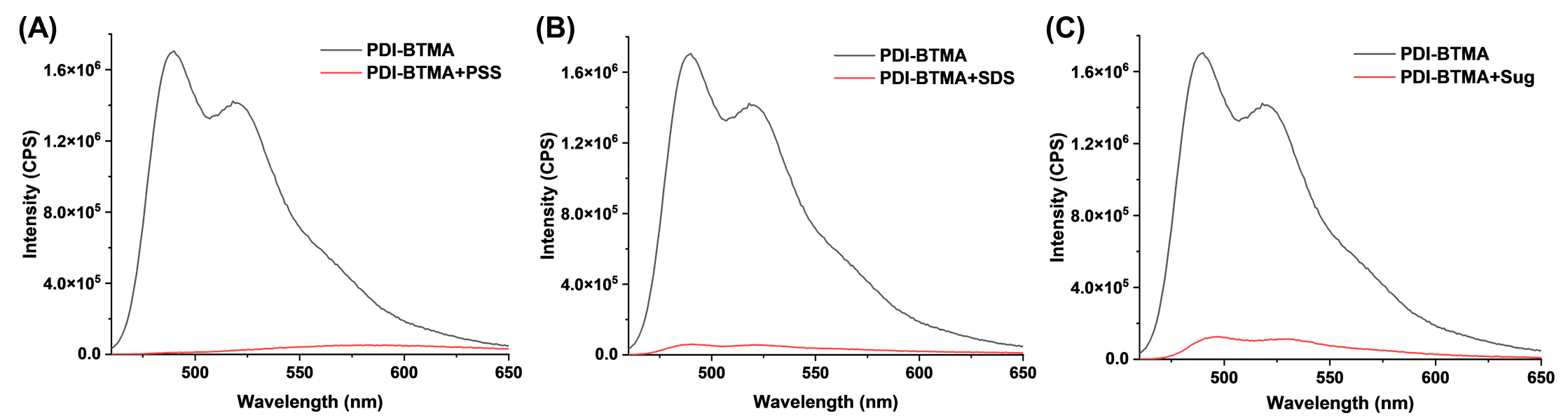


**Figure S12.** Fluorescence spectra of PDI-BTMA (1 μM) before and after assembly with PSS (0.01 μM)/SDS (30 μM)/Sug (1 μM) in HEPES buffer (10 mM, pH = 7), *λ*_ex_ = 440 nm.


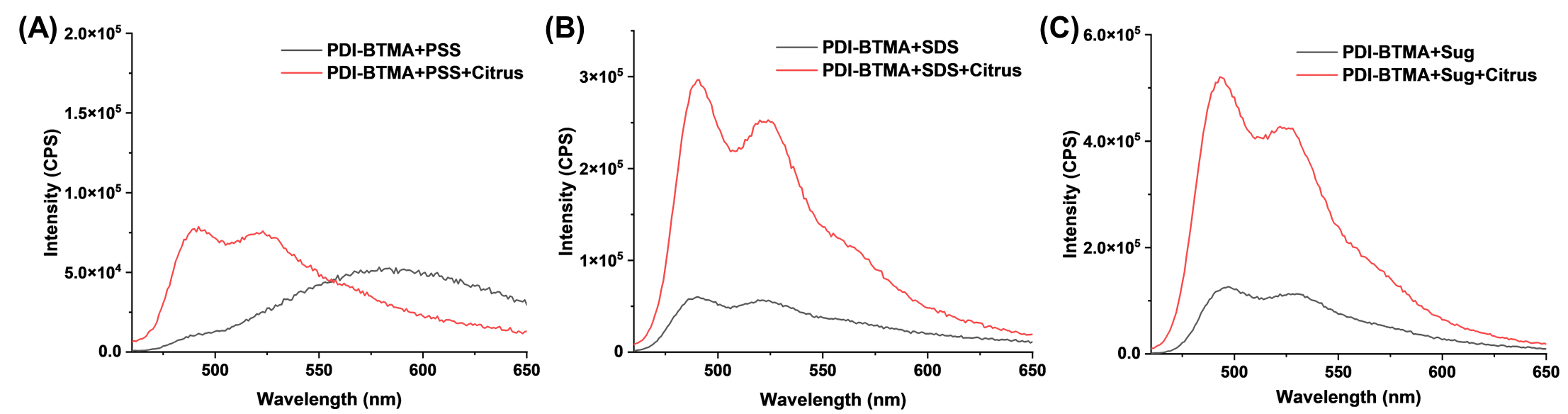


**Figure S13.** Fluorescence changes of sensor units in **sensor array 2** after the addition of citrus sample in HEPES buffer (10 mM, pH = 7), *λ*_ex_ = 440 nm.

**Table S3.** The training matrix of fluorescence response patterns of **sensor array 2** against different citrus samples.

|  | **SU 4** | **SU 5** | **SU 6** |
| --- | --- | --- | --- |
| South Africa | 0.5241 | 0.6400 | 1.4256 |
| South Africa | 0.5179 | 0.6435 | 1.4257 |
| South Africa | 0.5206 | 0.6258 | 1.4121 |
| South Africa | 0.5560 | 0.6037 | 1.4274 |
| South Africa | 0.5364 | 0.6314 | 1.4165 |
| Australia | -0.2594 | -0.4652 | 0.1162 |
| Australia | -0.2693 | -0.4755 | 0.1210 |
| Australia | -0.2765 | -0.4641 | 0.1299 |
| Australia | -0.2885 | -0.4734 | 0.1272 |
| Australia | -0.2797 | -0.4731 | 0.1272 |
| China HN | -0.3378 | 0.0282 | 1.0569 |
| China HN | -0.3482 | 0.0228 | 1.0562 |
| China HN | -0.3496 | 0.0190 | 1.0714 |
| China HN | -0.3403 | -0.0064 | 1.0632 |
| China HN | -0.3504 | 0.0054 | 1.0223 |
| China ZJ | 0.1646 | 0.7623 | 1.3732 |
| China ZJ | 0.1730 | 0.7420 | 1.3771 |
| China ZJ | 0.1416 | 0.7692 | 1.3757 |
| China ZJ | 0.1692 | 0.7655 | 1.3762 |
| China ZJ | 0.1613 | 0.7787 | 1.4190 |
| China GX | -0.4883 | -0.3108 | 0.0863 |
| China GX | -0.4915 | -0.3133 | 0.0696 |
| China GX | -0.4914 | -0.3151 | 0.0715 |
| China GX | -0.4761 | -0.3093 | 0.0767 |
| China GX | -0.4775 | -0.3136 | 0.0880 |
| China YN | 0.1842 | 0.4317 | 1.5324 |
| China YN | 0.1975 | 0.4102 | 1.5502 |
| China YN | 0.1940 | 0.3716 | 1.4895 |
| China YN | 0.2089 | 0.3950 | 1.5244 |
| China YN | 0.2146 | 0.3796 | 1.5181 |


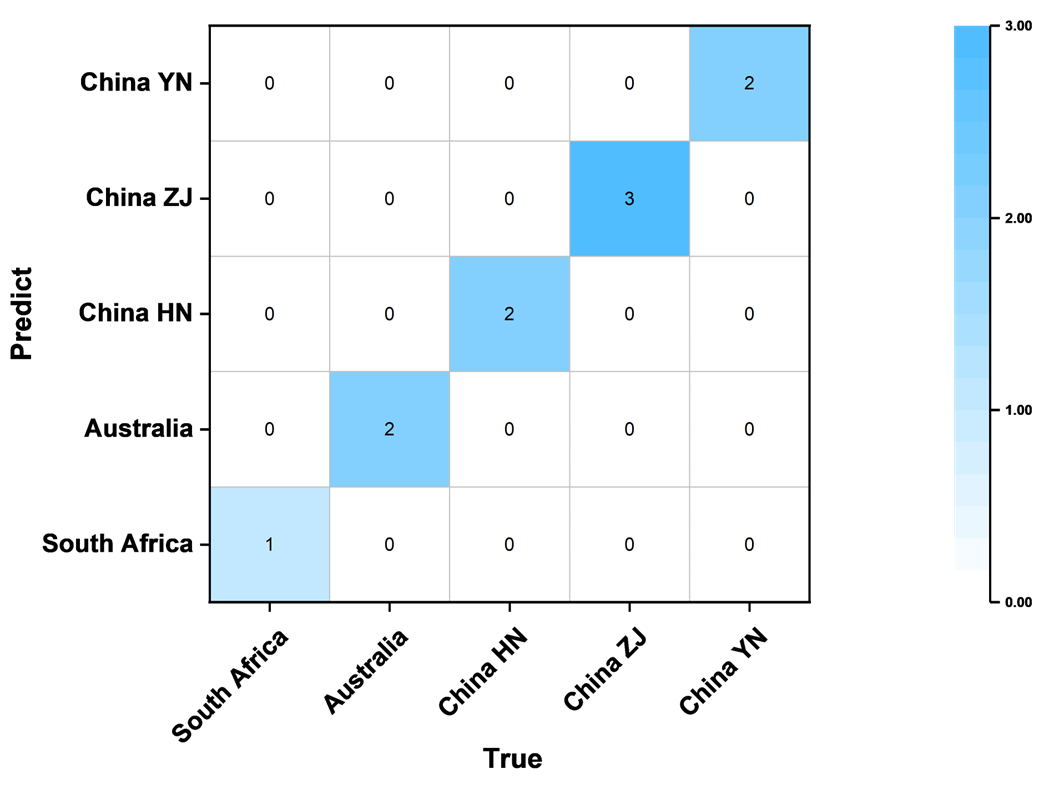


**Figure S14.** Confusion matrix heatmap for classification predictions of citrus regions.


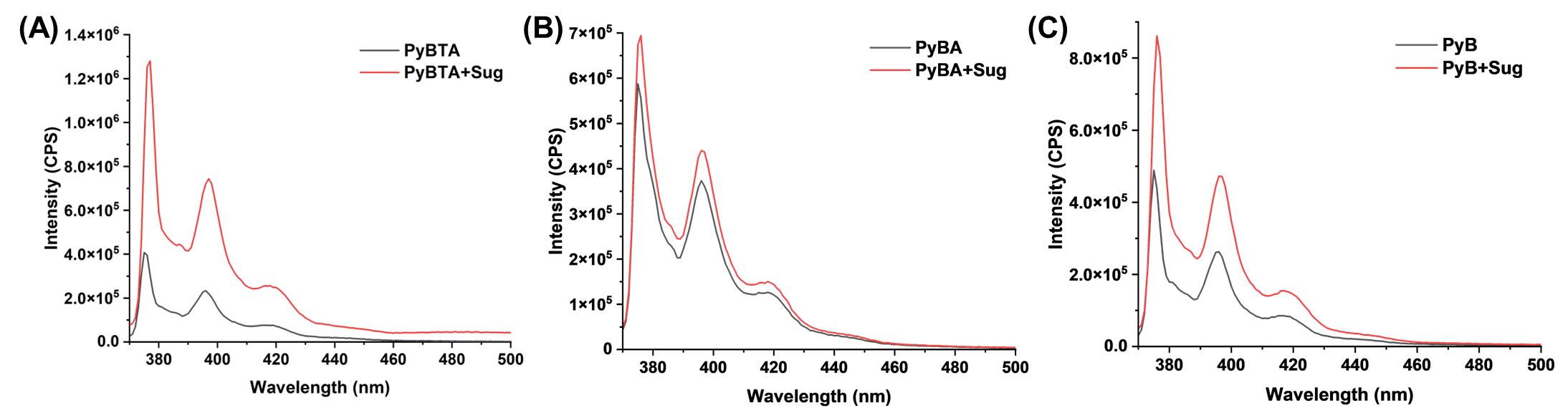


**Figure S15.** Fluorescence spectra of PyBTA/PyBA/PyB (5 μM) before and after assembly with Sug (10 μM) in HEPES buffer (10 mM, pH = 7), *λ*_ex_ = 350 nm.


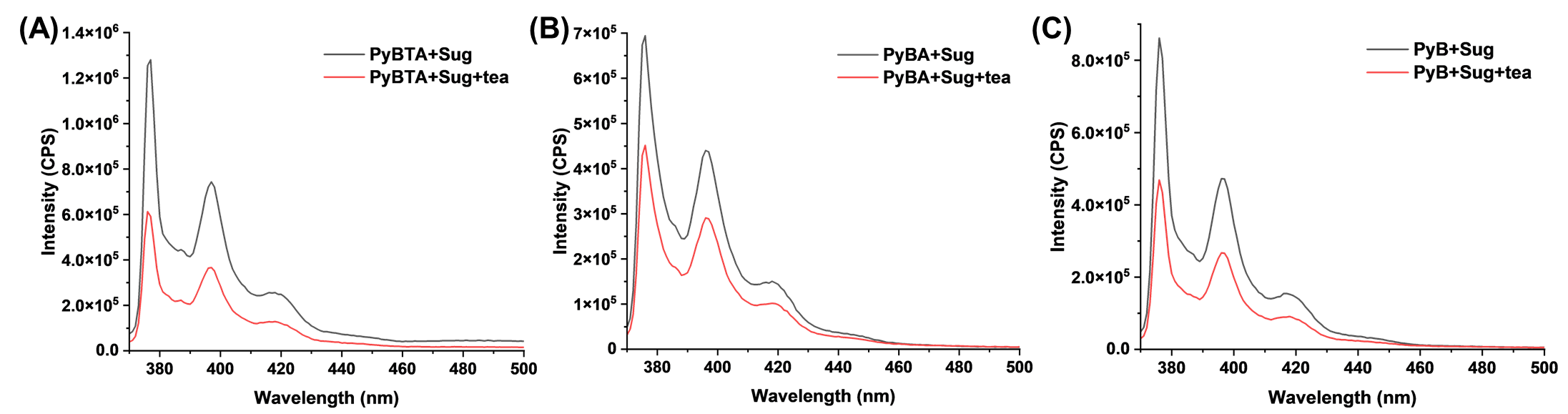


**Figure S16.** Fluorescence changes of sensor units in **sensor array 3** after the addition of tea sample in HEPES buffer (10 mM, pH = 7), *λ*_ex_ = 350 nm.

**Table S4.** The training matrix of fluorescence response patterns of **sensor array 3** against different types of green tea samples.

|  | **SU 3** | **SU 7** | **SU 8** |
| --- | --- | --- | --- |
| DTBLC | 0.2547 | 0.2108 | 0.3519 |
| DTBLC | 0.2576 | 0.1983 | 0.3547 |
| DTBLC | 0.2554 | 0.2042 | 0.3543 |
| DTBLC | 0.2579 | 0.2106 | 0.3479 |
| DTBLC | 0.2575 | 0.1996 | 0.3470 |
| HSMF | 0.2307 | 0.1883 | 0.3120 |
| HSMF | 0.2335 | 0.1891 | 0.3162 |
| HSMF | 0.2352 | 0.1829 | 0.3179 |
| HSMF | 0.2345 | 0.1955 | 0.3201 |
| HSMF | 0.2300 | 0.1828 | 0.3121 |
| LAGP | 0.4616 | 0.3910 | 0.5152 |
| LAGP | 0.4595 | 0.3888 | 0.5145 |
| LAGP | 0.4600 | 0.3922 | 0.5081 |
| LAGP | 0.4583 | 0.3916 | 0.5133 |
| LAGP | 0.4606 | 0.3911 | 0.5144 |
| LJ | 0.4948 | 0.4127 | 0.5498 |
| LJ | 0.4985 | 0.4115 | 0.5448 |
| LJ | 0.4962 | 0.4095 | 0.5526 |
| LJ | 0.4973 | 0.4076 | 0.5507 |
| LJ | 0.4965 | 0.4176 | 0.5510 |
| XYMJ | 0.3754 | 0.3316 | 0.4362 |
| XYMJ | 0.3791 | 0.3250 | 0.4426 |
| XYMJ | 0.3730 | 0.3315 | 0.4449 |
| XYMJ | 0.3797 | 0.3290 | 0.4371 |
| XYMJ | 0.3803 | 0.3306 | 0.4456 |
| XZZJ | 0.4000 | 0.3309 | 0.4693 |
| XZZJ | 0.3971 | 0.3417 | 0.4692 |
| XZZJ | 0.4000 | 0.3349 | 0.4751 |
| XZZJ | 0.3980 | 0.3375 | 0.4642 |
| XZZJ | 0.3969 | 0.3307 | 0.4745 |

**Table S5.** The training matrix of fluorescence response patterns of **sensor array 3** against different grades of Pu-erh tea samples.

|  | **SU 3** | **SU 7** | **SU 8** |
| --- | --- | --- | --- |
| Chunjian | 0.4065 | 0.3302 | 0.4655 |
| Chunjian | 0.4095 | 0.3283 | 0.4681 |
| Chunjian | 0.4084 | 0.3336 | 0.4723 |
| Chunjian | 0.4030 | 0.3312 | 0.4680 |
| Chunjian | 0.4083 | 0.3360 | 0.4657 |
| Maanshan | 0.2844 | 0.1291 | 0.3113 |
| Maanshan | 0.2757 | 0.1264 | 0.3192 |
| Maanshan | 0.2734 | 0.1251 | 0.3162 |
| Maanshan | 0.2747 | 0.1318 | 0.3089 |
| Maanshan | 0.2788 | 0.1347 | 0.3105 |
| Mangfei | 0.3743 | 0.2607 | 0.4153 |
| Mangfei | 0.3733 | 0.2573 | 0.4182 |
| Mangfei | 0.3686 | 0.2577 | 0.4170 |
| Mangfei | 0.3730 | 0.2590 | 0.4137 |
| Mangfei | 0.3763 | 0.2616 | 0.4126 |
| Yiwu | 0.3392 | 0.2333 | 0.3761 |
| Yiwu | 0.3396 | 0.2316 | 0.3734 |
| Yiwu | 0.3387 | 0.2305 | 0.3699 |
| Yiwu | 0.3359 | 0.2283 | 0.3705 |
| Yiwu | 0.3440 | 0.2288 | 0.3719 |
| Yanzitou | 0.0547 | 0.0412 | 0.1959 |
| Yanzitou | 0.0462 | 0.0354 | 0.1902 |
| Yanzitou | 0.0521 | 0.0291 | 0.1904 |
| Yanzitou | 0.0521 | 0.0305 | 0.1867 |
| Yanzitou | 0.0551 | 0.0284 | 0.1845 |


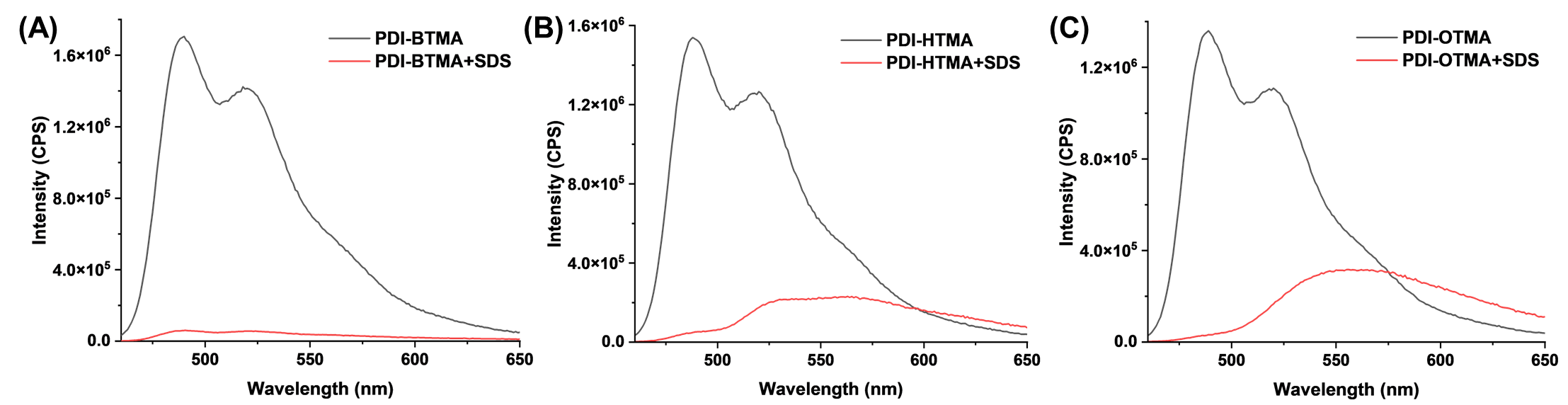


**Figure S17.** Fluorescence spectra of PDI-BTMA/PDI-HTMA/PDI-OTMA (1 μM) before and after assembly with SDS (30 μM) in HEPES buffer (10 mM, pH = 7), *λ*_ex_ = 440 nm.


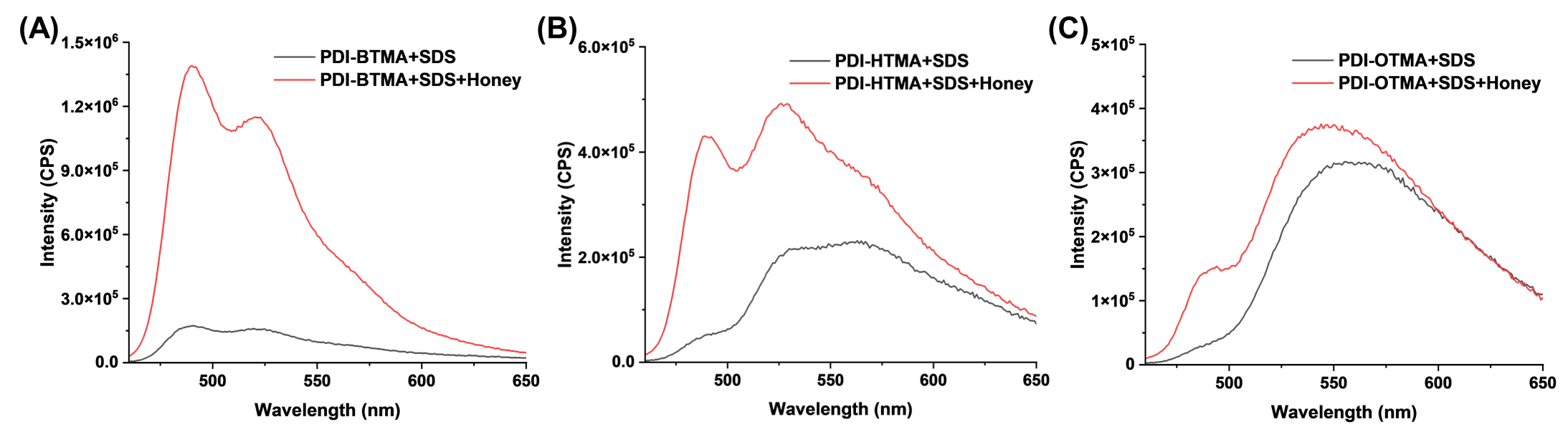


**Figure S18.** Fluorescence spectra of sensor units in **sensor array 4** in HEPES buffer (10 mM, pH = 7) and in the honey sample, *λ*_ex_ = 440 nm.


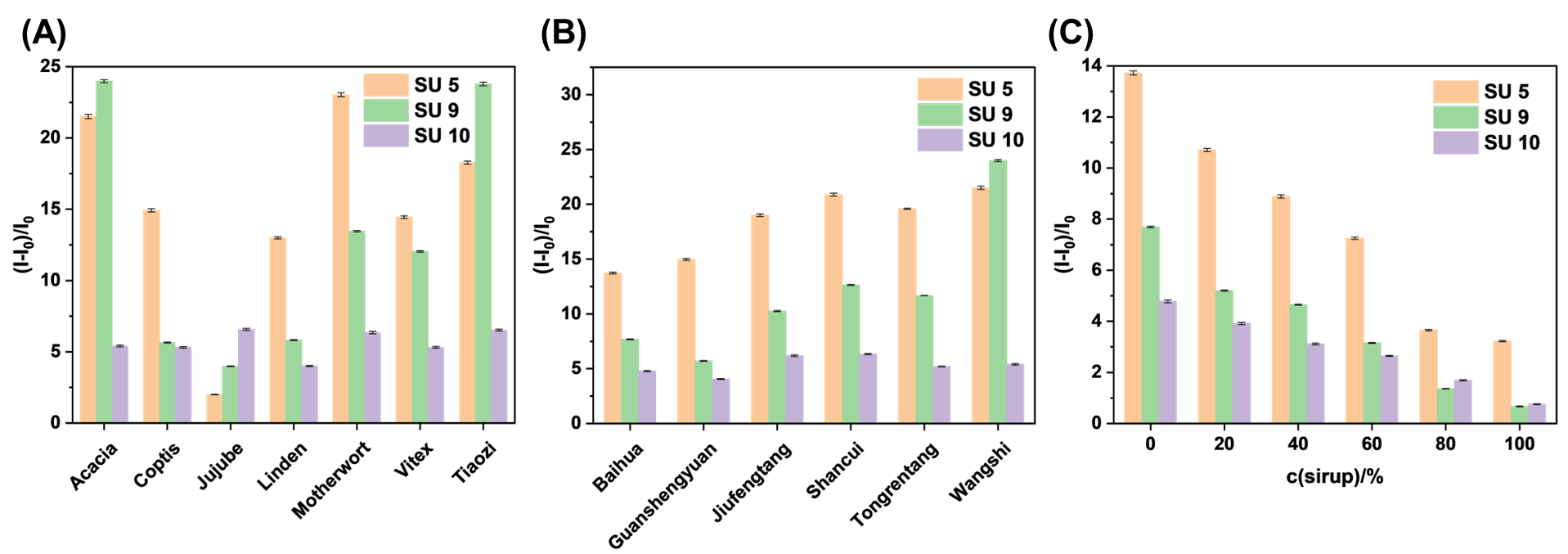


**Figure S19.** Fluorescence response patterns of **sensor array 4** to (A) seven types of honey from Wang’s brand, (B) six brands of acacia honey, and (C) honey adulterated with different proportions of sirup.

**Table S6.** The training matrix of fluorescence response patterns of **sensor array 4** against different types of honey samples.

|  | **SU 5** | **SU 9** | **SU 10** |
| --- | --- | --- | --- |
| Acacia | 21.4205 | 24.0785 | 5.4880 |
| Acacia | 21.4034 | 24.2021 | 5.1888 |
| Acacia | 22.0042 | 23.6746 | 5.5862 |
| Acacia | 21.1236 | 24.1172 | 5.3533 |
| Acacia | 21.5690 | 23.8347 | 5.3920 |
| Coptis | 14.8502 | 5.6335 | 5.4465 |
| Coptis | 14.8513 | 5.7208 | 5.1505 |
| Coptis | 15.2862 | 5.5370 | 5.4106 |
| Coptis | 14.5972 | 5.7139 | 5.2472 |
| Coptis | 15.0319 | 5.6174 | 5.2864 |
| Jujube | 1.9958 | 4.0275 | 6.6782 |
| Jujube | 2.0030 | 3.9881 | 6.4233 |
| Jujube | 2.0812 | 3.9739 | 6.8106 |
| Jujube | 1.9559 | 4.0249 | 6.4896 |
| Jujube | 2.0138 | 3.9394 | 6.4708 |
| Linden | 12.8867 | 5.8031 | 4.0212 |
| Linden | 12.9604 | 5.8711 | 3.9038 |
| Linden | 13.2458 | 5.7316 | 4.1072 |
| Linden | 12.7905 | 5.9071 | 3.9779 |
| Linden | 13.0800 | 5.7844 | 4.0028 |
| Motherwort | 22.9903 | 13.4018 | 6.3836 |
| Motherwort | 22.9691 | 13.6162 | 6.1244 |
| Motherwort | 23.4673 | 13.3222 | 6.6036 |
| Motherwort | 22.6592 | 13.5080 | 6.2227 |
| Motherwort | 23.0588 | 13.4550 | 6.3868 |
| Vitex | 14.3451 | 12.0598 | 5.3874 |
| Vitex | 14.4494 | 12.0964 | 5.1371 |
| Vitex | 14.7815 | 11.8960 | 5.4908 |
| Vitex | 14.1748 | 12.1791 | 5.2077 |
| Vitex | 14.5132 | 11.9874 | 5.3581 |
| Tiaozi | 18.2874 | 23.7530 | 6.5743 |
| Tiaozi | 18.2251 | 23.9512 | 6.3455 |
| Tiaozi | 18.6158 | 23.3368 | 6.6925 |
| Tiaozi | 17.9236 | 24.1189 | 6.4424 |
| Tiaozi | 18.3126 | 23.7603 | 6.5464 |

**Table S7.** The training matrix of fluorescence response patterns of **sensor array 4** against different brands of honey samples.

|  | **SU 5** | **SU 9** | **SU 10** |
| --- | --- | --- | --- |
| Baihua | 13.6089 | 7.7072 | 4.8770 |
| Baihua | 13.6358 | 7.7703 | 4.6571 |
| Baihua | 13.9832 | 7.5917 | 4.9341 |
| Baihua | 13.5807 | 7.7223 | 4.6467 |
| Baihua | 13.8142 | 7.6556 | 4.8094 |
| Guanshengyuan | 14.8714 | 5.6613 | 4.0841 |
| Guanshengyuan | 14.8751 | 5.7482 | 3.9677 |
| Guanshengyuan | 15.2351 | 5.6037 | 4.1821 |
| Guanshengyuan | 14.7397 | 5.8360 | 3.9966 |
| Guanshengyuan | 15.1179 | 5.6825 | 4.0751 |
| Jiufengtang | 18.9843 | 10.2975 | 6.2980 |
| Jiufengtang | 18.9160 | 10.3663 | 6.0231 |
| Jiufengtang | 19.4039 | 10.0723 | 6.4261 |
| Jiufengtang | 18.6963 | 10.3430 | 6.0647 |
| Jiufengtang | 19.0754 | 10.2078 | 6.1776 |
| Shancui | 20.7932 | 12.6550 | 6.4143 |
| Shancui | 20.8006 | 12.7003 | 6.1823 |
| Shancui | 21.2925 | 12.5352 | 6.4807 |
| Shancui | 20.5093 | 12.7570 | 6.2365 |
| Shancui | 21.0003 | 12.5496 | 6.3454 |
| Tongrentang | 19.5281 | 11.6669 | 5.2255 |
| Tongrentang | 19.5792 | 11.8590 | 5.0755 |
| Tongrentang | 19.9340 | 11.5303 | 5.3551 |
| Tongrentang | 19.2714 | 11.7165 | 5.1469 |
| Tongrentang | 19.6643 | 11.6473 | 5.2099 |
| Wangshi | 21.4205 | 24.0785 | 5.4880 |
| Wangshi | 21.4034 | 24.2021 | 5.1888 |
| Wangshi | 22.0042 | 23.6746 | 5.5862 |
| Wangshi | 21.1236 | 24.1172 | 5.3533 |
| Wangshi | 21.5690 | 23.8347 | 5.3920 |

**Table S8.** The training matrix of fluorescence response patterns of **sensor array 4** against honey samples adulterated with different proportions of sirup.

|  | **SU 5** | **SU 9** | **SU 10** |
| --- | --- | --- | --- |
| 0% | 13.6089 | 7.7072 | 4.8770 |
| 0% | 13.6358 | 7.7703 | 4.6571 |
| 0% | 13.9832 | 7.5917 | 4.9341 |
| 0% | 13.5807 | 7.7223 | 4.6467 |
| 0% | 13.8142 | 7.6556 | 4.8094 |
| 20% | 10.6474 | 5.2401 | 3.9776 |
| 20% | 10.6999 | 5.2627 | 3.8048 |
| 20% | 10.8941 | 5.1514 | 4.0065 |
| 20% | 10.5351 | 5.2191 | 3.8283 |
| 20% | 10.7773 | 5.1485 | 3.9544 |
| 40% | 8.9214 | 4.6714 | 3.1416 |
| 40% | 8.8304 | 4.7115 | 3.0395 |
| 40% | 9.0726 | 4.6018 | 3.2379 |
| 40% | 8.7030 | 4.6451 | 3.0538 |
| 40% | 8.9315 | 4.6295 | 3.1000 |
| 60% | 7.2123 | 3.2000 | 2.6777 |
| 60% | 7.2233 | 3.1796 | 2.5774 |
| 60% | 7.3771 | 3.0979 | 2.7099 |
| 60% | 7.1293 | 3.1383 | 2.6438 |
| 60% | 7.3404 | 3.1541 | 2.6226 |
| 80% | 3.6503 | 1.3573 | 1.6896 |
| 80% | 3.6366 | 1.3656 | 1.6644 |
| 80% | 3.7506 | 1.3380 | 1.7787 |
| 80% | 3.5749 | 1.3992 | 1.6482 |
| 80% | 3.6652 | 1.3534 | 1.6864 |
| 100% | 3.1645 | 0.6857 | 0.7442 |
| 100% | 3.2051 | 0.6797 | 0.7228 |
| 100% | 3.3182 | 0.6133 | 0.8104 |
| 100% | 3.1771 | 0.6943 | 0.7197 |
| 100% | 3.2495 | 0.6491 | 0.7718 |


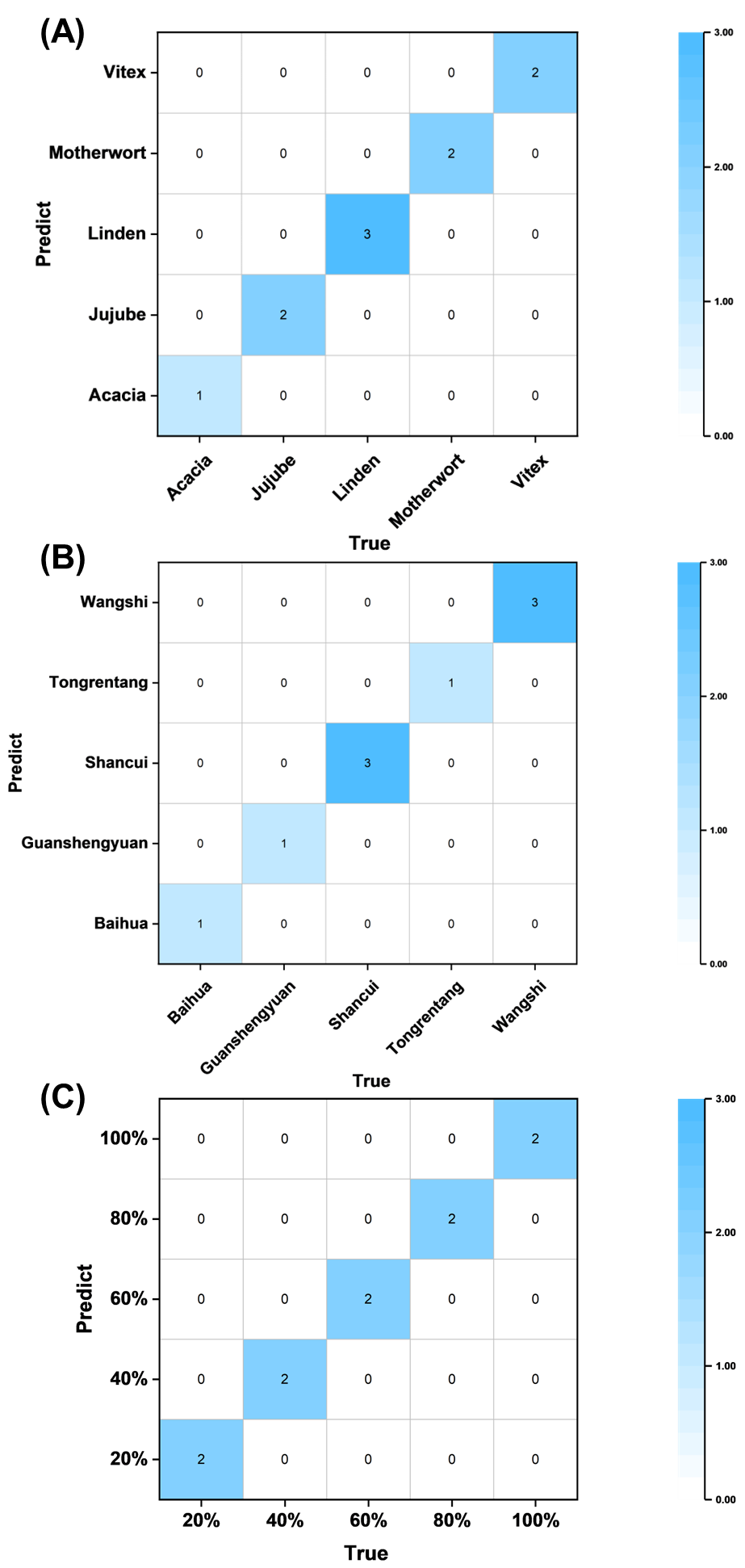


**Figure S20.** Confusion matrix heatmap for classification predictions of (A) honey types, (B) honey brands, and (C) honey adulterated with sirup.


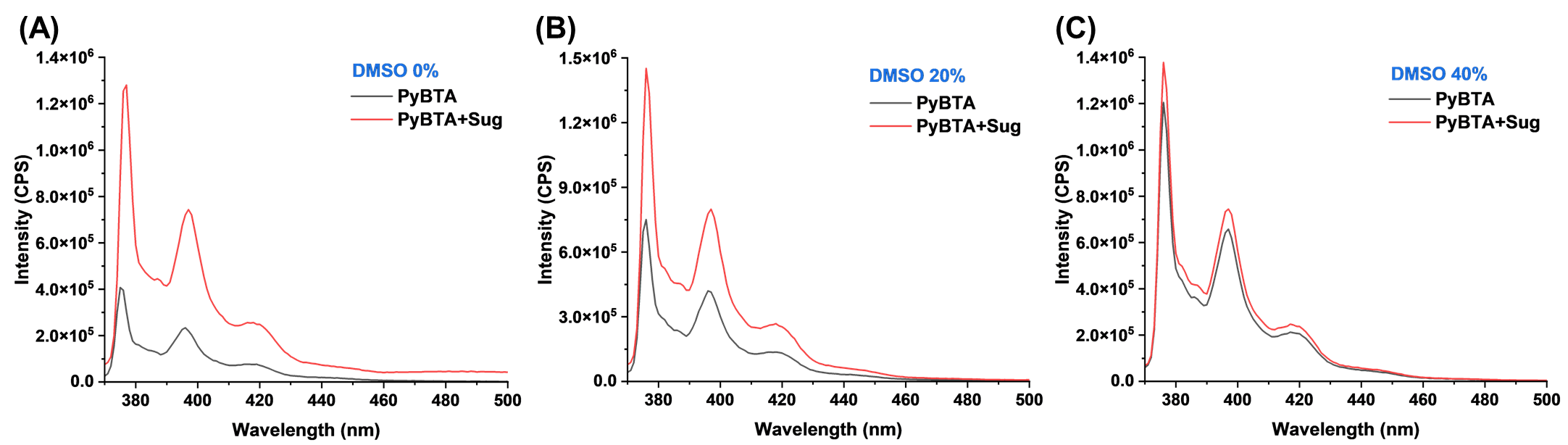


**Figure S21.** Fluorescence spectra of PyBTA (5 μM) before and after assembly with Sug (10 μM) in HEPES buffer containing 0%, 20%, and 40% DMSO, *λ*_ex_ = 350 nm.


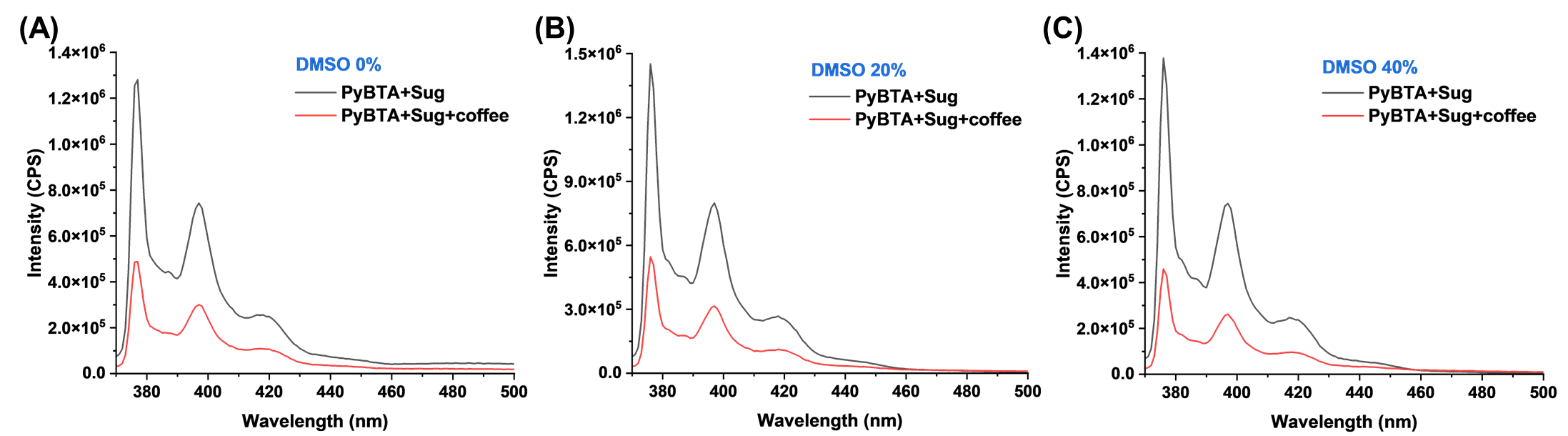


**Figure S22.** Fluorescence changes of sensor units in **sensor array 5** after the addition of coffee bean samples, *λ*_ex_ = 350 nm.

**Table S9.** The training matrix of fluorescence response patterns of **sensor array 5** against different coffee bean samples.

|  | **SU 3** | **SU 11** | **SU 12** |
| --- | --- | --- | --- |
| China | 0.5416 | 0.5583 | 0.5852 |
| China | 0.5381 | 0.5589 | 0.5874 |
| China | 0.5412 | 0.5578 | 0.5879 |
| China | 0.5397 | 0.5604 | 0.5823 |
| China | 0.5377 | 0.5617 | 0.5815 |
| Costa Rica | 0.5069 | 0.5068 | 0.5188 |
| Costa Rica | 0.5038 | 0.5060 | 0.5169 |
| Costa Rica | 0.5072 | 0.5076 | 0.5169 |
| Costa Rica | 0.5045 | 0.5055 | 0.5194 |
| Costa Rica | 0.5041 | 0.5069 | 0.5180 |
| Ethiopia | 0.6192 | 0.6048 | 0.6512 |
| Ethiopia | 0.6202 | 0.6020 | 0.6533 |
| Ethiopia | 0.6201 | 0.6048 | 0.6551 |
| Ethiopia | 0.6205 | 0.6025 | 0.6508 |
| Ethiopia | 0.6173 | 0.6043 | 0.6523 |
| Indonesia | 0.5443 | 0.5624 | 0.5507 |
| Indonesia | 0.5432 | 0.5574 | 0.5552 |
| Indonesia | 0.5467 | 0.5595 | 0.5564 |
| Indonesia | 0.5402 | 0.5551 | 0.5526 |
| Indonesia | 0.5457 | 0.5581 | 0.5561 |
| Kenya | 0.6034 | 0.6037 | 0.6384 |
| Kenya | 0.6028 | 0.6036 | 0.6402 |
| Kenya | 0.6037 | 0.6026 | 0.6369 |
| Kenya | 0.6009 | 0.6024 | 0.6347 |
| Kenya | 0.6002 | 0.6031 | 0.6366 |


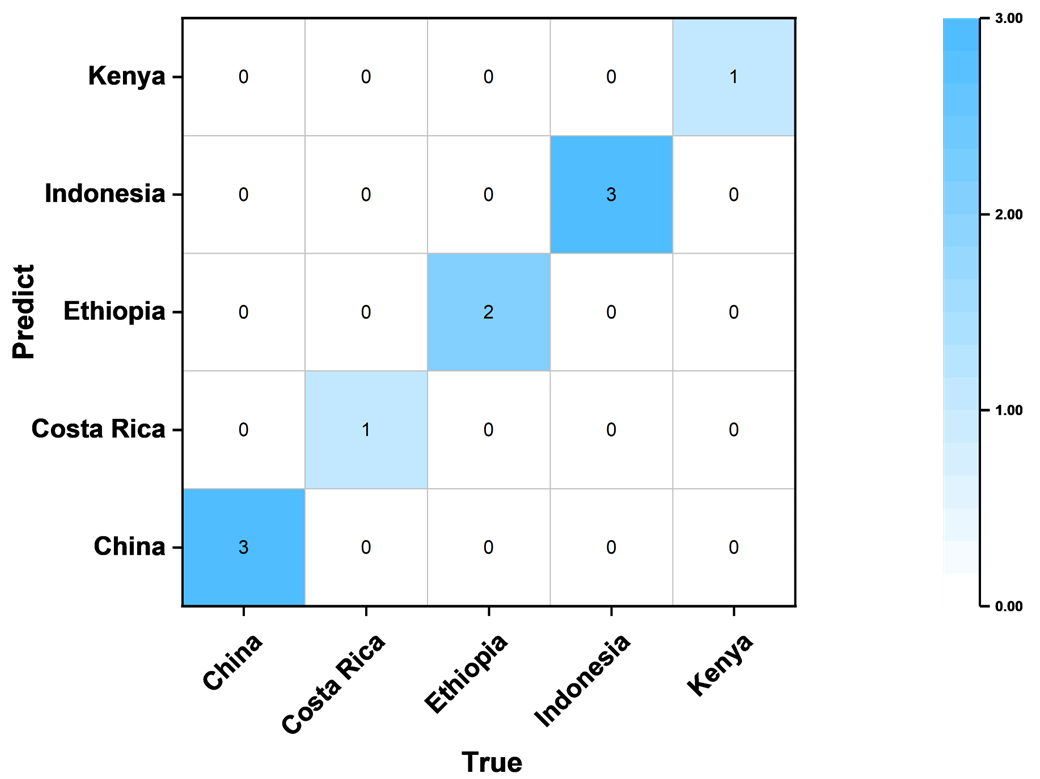


**Figure S23.** Confusion matrix heatmap for classification predictions of coffee regions.

**References**

[1] Z. Yao, Y. Qiao, H. Liang, W. Ge, L. Zhang, Z. Cao, H.-C. Wu, *Anal. Chem.* **2016**, *88*, 10605-10610.

[2] J. Chen, H. Jiao, W. Li, D. Liao, H. Zhou, C. Yu, *Chem.-Asian J.* **2013**, *8*, 276-281.
